# Social inequalities in child mental health trajectories: a longitudinal study using birth cohort data 12 countries

**DOI:** 10.1186/s12889-024-20291-5

**Published:** 2024-10-22

**Authors:** Tim Cadman, Demetris Avraam, Jennie Carson, Ahmed Elhakeem, Veit Grote, Kathrin Guerlich, Mònica Guxens, Laura D. Howe, Rae-Chi Huang, Jennifer R. Harris, Tanja A. J. Houweling, Eleanor Hyde, Vincent Jaddoe, Pauline W. Jansen, Jordi Julvez, Berthold Koletzko, Ashleigh Lin, Katerina Margetaki, Maria Melchior, Johanna Thorbjornsrud Nader, Marie Pedersen, Costanza Pizzi, Theano Roumeliotaki, Morris Swertz, Muriel Tafflet, David Taylor-Robinson, Robyn E. Wootton, Katrine Strandberg-Larsen

**Affiliations:** 1 Department of Public Health, Section of Epidemiology, University of Copenhagen, Copenhagen, Denmark; 2 Department of Public Health, Policy and Systems, University of Liverpool, Liverpool, UK; 3 Cardiovascular Epidemiology Research Centre, School of Population and Global Health, The University of Western Australia, Crawley, WA Australia; 4 MRC Integrative Epidemiology Unit, University of Bristol, Bristol, UK; 5 Division of Metabolic and Nutritional Medicine, Department of Pediatrics, Dr. Von Hauner Children’s Hospital, University Hospital, LMU Munich, Munich, Germany; 6 ISGlobal, Barcelona, Spain; 7 Universitat Pompeu Fabra, Barcelona, Spain; 8 Spanish Consortium for Research On Epidemiology and Public Health (CIBERESP), Instituto de Salud Carlos III, Madrid, Spain; 9 Department of Child and Adolescent Psychiatry/Psychology, Erasmus MC, University Medical Centre, Rotterdam, The Netherlands; 10 Nutrition & Health Innovation Research Institute, Edith Cowan University, Perth, Australia; 11 Centre for Fertility and Health, Norwegian Institute of Public Health, Oslo, Norway; 12 Department of Public Health, Erasmus MC, University Medical Center, Rotterdam, CA 3000 The Netherlands; 13 UMCG Genetics Department, University Medical Centre Groningen, Genetics Department (GCC ‐ Genomic Coordination Centre), Groningen, The Netherlands; 14 Department of Pediatrics, Erasmus MC University Medical Center, Rotterdam, The Netherlands; 15 The Generation R Study Group, Erasmus MC University Medical Center, Rotterdam, The Netherlands; 16 Department of Psychology, Education and Child Studies, Erasmus University Rotterdam, Rotterdam, The Netherlands; 17 Institut d’Investigació Sanitària Pere Virgili (IISPV), Clinical and Epidemiological Neuroscience Group (NeuroÈpia), Reus (Tarragona), Catalonia 43204 Spain; 18 School of Population and Global Health, University of Western Australia, Nedlands, Australia; 19 Department of Social Medicine, Medical School, Clinic of Preventive Medicine and Nutrition, University of Crete, Heraklion, Greece; 20 Sorbonne Université, INSERM, Institut Pierre Louis d’Epidémiologie Et de Santé Publique (IPLESP), Equipe de Recherche en Epidémiologie Sociale (ERES), Faculté de Médecine St Antoine, Paris, France; 21 Department of Genetics and Bioinformatics, Division of Health Data and Digitalisation, Norwegian Institute of Public Health, Oslo, Norway; 22 Department of Medical Sciences, Cancer Epidemiology Unit, University of Turin and CPO Piemonte, Turin, Italy; 23 Centre for Research in Epidemiology and StatisticS (CRESS), Inserm, INRAE, Université Paris Cité, Paris, France; 24 School of Psychological Science, University of Bristol, UK, and Nic Waals Institute, Lovisenberg Hospital, Oslo, Norway

**Keywords:** Internalising problems, Externalising problems, Socio‐economic circumstances, Socio‐economic position, Trajectories, Social inequalities, Child mental health

## Abstract

**Background:**

Social inequalities in child mental health are an important public health concern. Whilst previous studies have examined inequalities at a single time point, very few have used repeated measures outcome data to describe how these inequalities emerge. Our aims were to describe social inequalities in child internalising and externalising problems across multiple countries and to explore how these inequalities change as children age.

**Methods:**

We used longitudinal data from eight birth cohorts containing participants from twelve countries (Australia, Belgium, Denmark, France, Germany, Greece, Italy, Netherlands, Poland, Norway, Spain and the United Kingdom). The number of included children in each cohort ranged from *N* = 584 (Greece) to *N* = 73,042 (Norway), with a total sample of *N* = 149,604. Child socio‐economic circumstances (SEC) were measured using self‐reported maternal education at birth. Child mental health outcomes were internalising and externalising problems measured using either the Strengths and Difficulties Questionnaire or the Child Behavior Checklist. The number of data collection waves in each cohort ranged from two to seven, with the mean child age ranging from two to eighteen years old. We modelled the slope index of inequality (SII) using sex‐stratified multi‐level models.

**Results:**

For almost all cohorts, at the earliest age of measurement children born into more deprived SECs had higher internalising and externalising scores than children born to less deprived SECs. For example, in Norway at age 2 years, boys born to mothers of lower education had an estimated 0.3 (95% CI 0.3, 0.4) standard deviation higher levels of internalising problems (SII) compared to children born to mothers with high education. The exceptions were for boys in Australia (age 2) and both sexes in Greece (age 6), where we observed minimal social inequalities. In UK, Denmark and Netherlands inequalities decreased as children aged, however for other countries (France, Norway, Australia and Crete) inequalities were heterogeneous depending on child sex and outcome. For all countries except France inequalities remained at the oldest point of measurement.

**Conclusions:**

Social inequalities in internalising and externalising problems were evident across a range of EU countries, with inequalities emerging early and generally persisting throughout childhood.

**Supplementary Information:**

The online version contains supplementary material available at 10.1186/s12889-024-20291-5.

## Introduction

Whilst it is well‐established that children born to disadvantaged socio‐economic circumstances (SEC) generally have worse mental health outcomes [1], most studies have assessed mental health inequalities at only a single time point [2]. Studies using repeated measures outcome data are important to establish the age at which inequalities in child mental health emerge and how they change during childhood [3, 4]. Identifying which outcomes show the most persistent inequalities, the ages at which inequalities emerge and the patterns of change over time can provide targets for policy and intervention.

Longitudinal research into child health inequalities is well established for growth measurements such as body‐mass index BMI and height. [5–9] Whilst studies focusing on mental health have reported higher levels of internalising (sadness, low mood) and externalising problems (behavioural problems and hyperactivity) in children from more deprived SEC as young as three years old, [10–13] only a handful of studies have modelled changes in inequalities, focusing on a few countries (such as the United Kingdom, Netherlands and Australia) and narrow age periods. Findings are inconsistent, with two studies reporting widening inequalities in internalising and externalising problems [14, 15], one study finding inequalities to remain constant [3] and two reporting a narrowing of inequalities [16, 17]. There is some evidence that inequalities in internalising problems may be smaller than for externalising problems, [18, 19] but again there is limited research on the course of these inequalities as children age.

The EU Child Cohort Network (ECCN) [20] contains harmonised data from multiple (mostly European) birth cohorts and provides a unique opportunity to examine mental health inequalities across different social contexts and over a wider age range than previously explored. In this study we focus on internalising and externalising problems, two key dimensions of children’s mental health associated with long‐term psychosocial outcomes [21] which are widely measured in birth cohort studies. We use the highest level of maternal education qualifications at birth as our indicator of child SEC as it is strongly related to income and employment, and also reflects non‐material family resources (e.g. knowledge) [22].

Our aims were to use data from twelve countries to describe social inequalities in internalising and externalising problems during childhood across different contexts, and to describe how these inequalities change as children age.

## Methods

### Inclusion criteria and participating cohorts

Pregnancy and birth cohort studies from the ECCN were eligible if the study contained data on maternal education, maternal age at birth, child sex and data on child internalising or externalising problems, where these were measured using the same instrument at a minimum of two data collection waves. We excluded cohorts that measured mental health only at a single measurement wave, or cohorts which measured problems at multiple waves but lacked measurements at two or more waves using the same instrument.

Eight cohorts had available data: Avon Longitudinal Study of Parents and Children (ALSPAC, United Kingdom), [23, 24] European Childhood Obesity Project Trial (CHOP; Germany, Belgium, Italy, Poland & Spain), [25] Danish National Birth Cohort (DNBC, Denmark), [26] Etude sur les. Déterminants de la santé de l’Enfant Nancy & Poitieres (EDEN‐Nancy & EDEN Poitiers, France), [27] Generation R (GenR, Netherlands), [28] Norwegian Mother, Father and Child Cohort (MoBa, Norway), [29] Generation 1 and Generation 2 participants of the Raine study (Raine, Australia), [30] and Rhea (Crete, Greece). [31] Further details of each cohort can be found in ECCN cohort profiles [20, 32] and each cohort’s profile paper. Individual participants from these cohorts were included if data were available on maternal education, maternal age at birth, sex, and at least one measurement of either internalising or externalising problems. The number of included children ranged from *N* = 584 (Rhea) to *N* = 73,042 (MoBa), with a total sample of *N* = 149,604 (Table 1).

**Table 1:** Distribution of mental health outcomes at each data collection wave

	Wave1		Wave2		Wave3		Wave4		Wave5		Wave6		Wave7	
	*N*	Age	*N*	Age	*N*	Age	*N*	Age	*N*	Age	*N*	Age	*N*	Age
ALSPAC (*N* = 9707)														
Internalising	8453	4 ± 0.1	7258	6.8 ± 0.2	6876	8.2 ± 0.1	6525	9.6 ± 0.1	5929	11.7 ± 0.1	5775	13.2 ± 0.2	4491	16.8 ± 0.3
Externalising	8428	4 ± 0.1	7463	6.8 ± 0.2	6902	8.2 ± 0.1	6597	9.6 ± 0.1	6054	11.7 ± 0.1	5941	13.2 ± 0.2	4613	16.8 ± 0.3
CHOP (*N* = 662)														
Internalising	574	5.5 ± 0.1	514	11.1 ± 0.1										
Externalising	574	5.5 ± 0.1	514	11.1 ± 0.1										
DNBC (*N* = 55163)														
Internalising	42410	7 ± 0	35596	11.2 ± 0.4	33447	18 ± 0								
Externalising	42380	7 ± 0	35596	11.2 ± 0.4	33447	18 ± 0								
EDEN (*N* = 1426)														
Internalising	1302	3.2 ± 0.1	1175	5.6 ± 0.2	872	8.1 ± 0.1								
Externalising	1302	3.2 ± 0.1	1175	5.6 ± 0.2	872	8.1 ± 0.1								
GenR (*N* = 6726)														
Internalising	5539	1.9 ± 0.7	2283	3.1 ± 0.2	4775	6.1 ± 0.5	3598	9.7 ± 0.3						
Externalising	5906	1.9 ± 0.7	2436	3.1 ± 0.2	4923	6.1 ± 0.5	3167	9.7 ± 0.3						
MoBa (*N* = 73042)														
Internalising	63929	1.5 ± 0	51271	3.1 ± 0.1	23612	5.2 ± 0.2								
Externalising	62613	1.5 ± 0	50676	3.1 ± 0.1	36128	5.2 ± 0.2								
The Raine study (*N* = 2294)														
Internalising	1661	2.1 ± 0.1	1809	5.9 ± 0.2	1730	8.1 ± 0.3	1646	10.6 ± 0.2						
Externalising	1677	2.1 ± 0.1	1945	5.9 ± 0.2	1815	8.1 ± 0.3	1707	10.6 ± 0.2						
Rhea (*N* = 584)														
Internalising	563	6.6 ± 0.3	332	11 ± 0.3										
Externalising	563	6.6 ± 0.3	332	11 ± 0.3										

Sample comprises participants with complete data on maternal education, child sex, maternal age at birth and at least one measurement of either internalising or externalising at one time point. ’N’ represents number of participants, ’Age’ is mean ± SD

### Exposure: maternal education

Maternal education at birth was harmonised in each cohort based on the International Standard Classification of Education 97 (ISCED‐97) and consisted of three categories: Low (No education to lower secondary; ISCED‐97 categories 0‐2), Medium (Upper and post‐secondary; ISCED‐97 categories 3‐4), and High (Degree and above; ISCED‐97 categories 5‐6) [33]. This was coded as a rank score (0‐1, with 1 representing low education) by creating a rank range based on the proportion of individuals within each category of maternal education and assigning each participant the midpoint of that rank [34]. For example, if 10% of mothers had high education, 40% medium and 50% low, the rank ranges would be high = 0 ‐ 0.1, medium = 0.1‐0.5 and low = 0.5 ‐ 1, with each participant assigned the midpoint within this range (0.05, 0.30, 0.75). [3]. Full details of the harmonisation process for all cohorts are provided at https://data-catalogue.molgeniscloud.org.

### Outcomes

We chose two domains reflecting key aspects of children’s mental health: internalising and externalising problems. The mean child age of data collection for each cohort is shown in Table 1. Internalising and externalising problems were assessed using either the Strengths and Difficulties Questionnaire (SDQ; ALSPAC, CHOP, DNBC & EDEN) [35] or the Child Behaviour Checklist (CBCL; GenR, MoBa & Rhea) [36]. The SDQ is a 25‐item questionnaire measured on a 3‐point Likert scale (“not true”, “somewhat true”, “certainly true”) containing five subscales: Emotional Problems, Conduct Problems, Hyperactivity/inattention, Peer problems and Pro‐social behaviour. An internalising score was calculated by summing the scores on the Emotional Problems and Peer Problems scales, whilst an externalising score was calculated by summing scores on the Conduct Problems and Hyperactivity subscales.

The CBCL/6‐18 is a 113‐item questionnaire rated on a 3‐point Likert scale (“not true”, “sometimes true”, “often true”), containing 8 subscales: Rule‐breaking Behavior, Aggressive Behavior, Withdrawn/Depressed, Somatic Complaints, Anxious/Depressed, Social Problems, Thought Problems and Attention Problems. An internalising score was calculated by summing the scores on the Withdrawn/Depressed, Somatic Complaints, and Anxiety/Depressed Problems subscales. From ages 5+, an externalising score was calculated by summing scores on Rule‐breaking Behavior and Aggressive Behavior, whilst at ages 1.5–5 years (CBCL/1½‐5, 99 items) it was calculated by summing score on the Aggressive Behavior and Attention Problems scales.

Parented‐report questionnaires were used for all cohorts and ages except the third data collection wave of the DNBC where self‐reported questionnaires were used (mean age 18 years). All outcomes were positively skewed, so to approximate a normal distribution a square‐root transformation was applied. To allow mental health outcomes to be compared on the same scale (rather than the original scale of the different instruments) internal z‐scores were calculated using the within‐cohort mean and standard deviation at each data collection wave.

### Confounders

Whilst there are several risk factors for mental health outcomes, few of these are hypothesised to cause maternal education. In the main analyses we adjusted only for maternal age at birth. Two other variables fit our definition of confounders but were not available in all cohorts: Maternal pre‐pregnancy mental health problems and maternal ethnicity. The presence of maternal pre‐pregnancy mental health problems (yes/no) were measured by self‐report and were available in six out of eight cohorts (all cohorts except CHOP and the Raine study). Maternal ethnicity (harmonised within the ECCN as Western vs Non‐Western) was available with sufficient variability (cell count non-western > 20) in three out of eight cohorts (ALSPAC, GenR & the Raine study). We therefore conducted sensitivity analyses additionally adjusting for these variables where available.

### Statistical analysis

All analyses were performed using DataSHIELD (R packages dsBaseClient 6.1.0 & dsHelper v1.1.0) a software solution which enables the federated analysis of data without the data being transferred and without researchers being able to view participant‐level data [37].

We fit multilevel models in each cohort with a random intercept for child and the following fixed effects: rank maternal education score, two fractional polynomial transformations of child age at mental health measurement (days) to account for non‐linear patterns over time, maternal age (years) and the interactions between the child age terms and maternal education (model equations described in Supplementary Text 2). To identify the model which best accounted for non‐linear change, for each outcome we tested multiple models including (up to two) transformations of the age‐term. We selected the model with the average lowest negative log‐likelihood across all cohorts [38], which for both outcomes was the model containing two age polynomials (child age^‐2^ and child age^‐1^). Preliminary analyses showed that polynomial models were a poor fit for two small cohorts with only two data collection waves (CHOP & Rhea), therefore for these cohorts we fit linear models containing fixed effect terms of rank maternal education, child age and the interaction between maternal education and age.

The coefficient of the rank score of maternal education is the Slope Index of Inequality (SII), which is the mean difference in outcome between the highest and lowest levels of maternal education assuming an underlying continuous distribution. As internalising and externalising scores were transformed to z‐scores, the SII represents the standard deviation (SD) difference in mental health between highest and lowest levels of education. The coefficient for the interaction between maternal education and the age term(s) gives information about the change in SII over time. Baseline inequalities and change in inequalities over time were described using model predicted SII at selected time points which included the first ages of measurement within each cohort (ages 2 to 8, 10, 12, 15 & 18 years).

As there is evidence that associations between SEP and mental health may differ by sex [3], we explored interactions by sex. We compared the fit of the model described above with a model which additionally contained fixed effects of (i) child sex, (ii) the 2‐way interaction between sex and maternal education, and (iii) the 3‐way interaction between sex, maternal education and each of the two age polynomial terms. Loglikehood tests showed that models including these interaction terms had a statistically better fit for 14 out of 16 comparisons (results not shown); therefore we present sex‐stratified analyses in the main results.

### Sensitivity analyses

To test the linearity of the association between maternal education and mental health outcomes, we plotted the trajectories of internalising and externalising across each level of maternal education. To evaluate whether results differed when using a different indicator of childhood SECs, we repeated analyses using household disposable income as the exposure (available in all cohorts except the Raine Study). The Equivalised Household Income Indicator (EHII) is an indicator of the total disposable monthly household income, standardized for the household size and composition. [39]. Disposable income was categorised into within‐cohort quintiles and also recoded as a rank score. We also repeated analyses additionally adjusting for maternal pre‐pregnancy mental health problems and ethnicity as described above.

### Missing data

Differences between the study sample (data on minimum maternal education, maternal age at birth and outcome at one time point) and the samples of excluded participants from each cohort are described in Supplementary Tables 1 and 2. Missing outcome data at all waves were handled using full information maximum likelihood estimation (FIML), which infers the values of missing data based on the distribution of available data. This assumes that the probability of an individual missing a measure of internalising or externalising does not depend on their underlying internalising or externalising problems score at that occasion, given their observed mental health symptoms at other occasions [40].

## Results

### Participants’ characteristics

There were considerable differences between cohorts in the level of maternal education, with the percentage of mothers with the lowest level of education ranging from 1.9 (MoBa) to 17.8 (CHOP; Table 2). For cohorts which recorded ethnicity, the majority of mothers had a Western background (> = 91%), with the exception of GenR which contained 64% of mothers of Western background and 36% mothers with a non‐Western background (Table 2).

**Table 2:** Descriptive statistics for socioeconomic exposures and covariates at birth

		ALSPAC (*N* = 9707)	CHOP (*N* = 662)	DNBC (*N* = 55,163)	EDEN (*N* = 1426)	GenR (*N* = 6726)	MoBa (*N* = 73,042)	The Raine study (*N* = 2294)	Rhea (*N* = 584)
Maternal Education	High	1420 (14.6)	206 (31.1)	28,018 (49.4)	840 (58.9)	3328 (49.5)	49,404 (67.6)	204 (34.9)	470 (20.5)
	Medium	6733 (69.4)	338 (51.1)	21,071 (37.1)	519 (36.4)	2863 (42.6)	22,250 (30.5)	299 (51.2)	627 (27.3)
	Low	1554 (16)	118 (17.8)	7666 (13.5)	67 (4.7)	535 (7.95)	1388 (1.9)	81 (13.9)	1197 (52.2)
Disposable Income	1st quintile	1418 (16.3)	79 (12.5)	9362 (18.6)	191 (13.9)	853 (15.5)	12,553 (20)	100 (22)	0 (NaN)
	2nd quintile	1702 (19.6)	102 (16.2)	10,098 (20.1)	271 (19.7)	1030 (18.7)	12,541 (20)	103 (22.6)	0 (NaN)
	3rd quintile	1845 (21.2)	133 (21.1)	10,233 (20.4)	301 (21.8)	1177 (21.4)	12,690 (20.2)	93 (20.4)	0 (NaN)
	4th quintile	1857 (21.3)	157 (24.9)	10,240 (20.4)	295 (21.4)	1207 (21.9)	12,482 (19.9)	105 (23.1)	0 (NaN)
	5th quintile	1879 (21.6)	160 (25.4)	10,315 (20.5)	320 (23.2)	1247 (22.6)	12,471 (19.9)	54 (11.9)	0 (NaN)
	Missing	1006 (10.4)	31 (4.68)	6507 (11.5)	48 (3.37)	1212 (18)	10,305 (14.1)	129 (22.1)	2294 (100)
Child sex	Male	4990 (51.4)	313 (47.3)	28,244 (49.8)	743 (52.1)	3386 (50.3)	37,400 (51.2)	327 (56)	1177 (51.3)
	Female	4717 (48.6)	349 (52.7)	28,511 (50.2)	683 (47.9)	3340 (49.7)	35,642 (48.8)	257 (44)	1117 (48.7)
Maternal age at birth	Mean ± SD	28.8 ± 4.62	30.9 ± 4.56	30.1 ± 4.21	30 ± 4.74	31.2 ± 4.93	30.4 ± 4.42	30 ± 4.77	28 ± 5.82
Maternal ethnicity	Western	9464 (98.7)	NA*	NA	NA*	4285 (63.8)	NA	NA*	2059 (89.8)
	Non‐western	129 (1.34)	NA*	NA	NA*	2435 (36.2)	NA	NA*	235 (10.2)
	Missing	114 (1.17)	NA*	NA	NA*	6 (0.09)	NA	NA*	0 (0)
Maternal mental health	No	8305 (89)	0 (NaN)	50,774 (92.9)	1357 (95.2)	3104 (69.8)	65,873 (92.2)	511 (97.3)	0 (NaN)
	Yes	1023 (11)	0 (NaN)	3903 (7.14)	69 (4.84)	1340 (30.2)	5594 (7.83)	14 (2.67)	0 (NaN)
	Missing	379 (3.9)	662 (100)	2078 (3.66)	0 (0)	2282 (33.9)	1575 (2.16)	59 (10.1)	2294 (100)
Internalising problems	Mean ± SD	2.6 ± 2.58	3.16 ± 2.73	3.31 ± 3.13	3.4 ± 2.64	5.64 ± 4.88	2.17 ± 2.09	6.6 ± 5.02	7.18 ± 5.67
Externalising problems	Mean ± SD	4.51 ± 3.25	5.34 ± 3.32	3.92 ± 3.07	5.73 ± 3.72	8.36 ± 6.32	5.04 ± 3.25	8.1 ± 6.48	9.92 ± 7.41

Figures represent N (%) for categorical variables and mean and standard deviation for continuous variables. Sample comprises participants with complete data on maternal education, child sex, maternal age at birth and at least one measurement of either internalising or externalising at one time point. ’N’ represents number of participants. NA* indicates cell counts <20 removed to prevent disclosure. CHOP, EDEN & Rhea excluded from ethnicity analysis

### Inequalities in child mental health by maternal education at first measurement wave

**Fig. 1:**
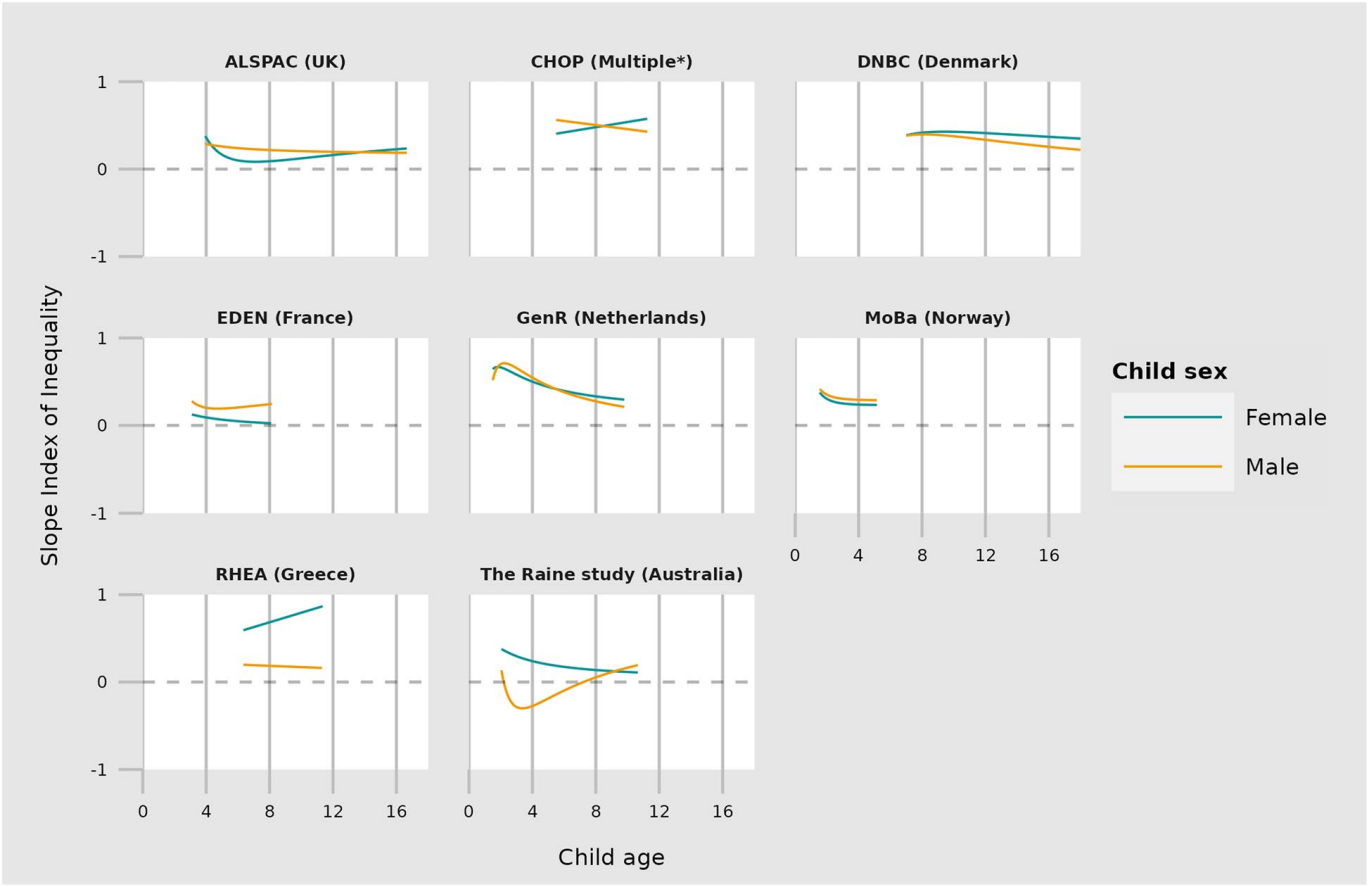
The SII (slope index of inequality) is plotted against age for each outcome. All outcomes are standardised to have a mean of zero and a variance of one. The SII therefore represents the mean difference in SDs of the outcome between the highest and lowest maternal education. *CHOP study includes data from Germany, Belgium, Italy, Poland \& Spain

**Fig. 2:**
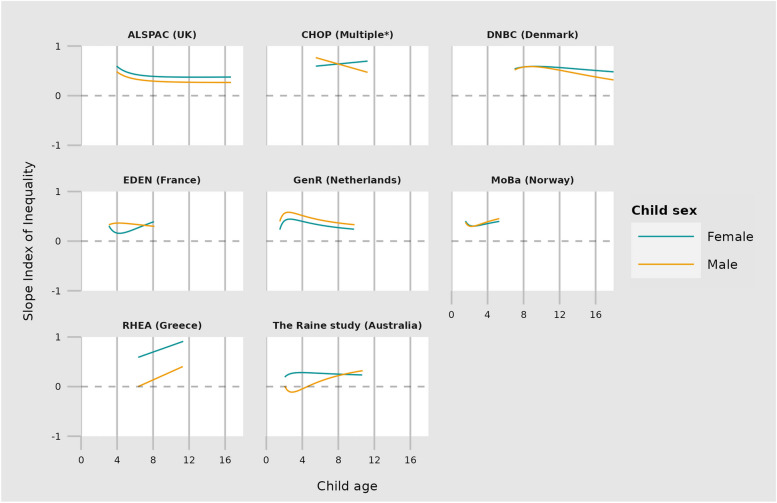
The SII (slope index of inequality) is plotted against age for each outcome. All outcomes are standardised to have a mean of zero and a variance of one. The SII therefore represents the mean difference in SDs of the outcome between the highest and lowest maternal education. *CHOP study includes data from Germany, Belgium, Italy, Poland \& Spain

Inequalities in internalising and externalising trajectories are depicted in Figs. 1 and 2, with model‐predicted SII at different ages described in Supplementary Tables 3 to 6. Most cohorts showed inequalities in both internalising and externalising problems at the earliest age of measurement, with children born to mothers of low education having more internalising and externalising problems than those born to mothers of high education. For example, in GenR at age 2 years the SII for internalising problems was 0.7 SD (95% CI 0.5, 0.8) for both girls and boys, and for externalising problems 0.4 SD for girls (95% CI 0.2, 0.6) and 0.6 SD for boys (95% CI 0.4, 0.7). Estimates for MoBa at two years of age were of smaller magnitude but in the same direction. For other cohorts children born to mothers with low vs high education also had more internalising and externalising problems (range 0.1 and 0.7 SD for both sexes and outcomes), except boys in the Raine study where lower maternal education was associated with fewer internalising problems.

### Change in inequalities in child mental health with age

We observed heterogeneity between cohorts. For both internalising and externalising problems, ALSPAC (ages 4‐16), GenR (ages 2‐9) & DNBC (ages 8‐18) showed decreasing inequalities as children aged. CHOP (ages 6‐11) showed decreasing inequalities for males but increasing for females for both internalising and externalising problems. MoBa showed decreasing inequalities for internalising but increasing for externalising, whilst trajectories for Eden, the Raine study and Rhea were heterogeneous between outcome and child sex. Whilst the trajectories of inequalities differed between cohorts, for all cohorts except EDEN (internalising, girls) inequalities remained at the oldest age of measurement. For example, in the two cohorts with the widest age range (ALSPAC & DNBC), inequalities at age 15 and 18 were between 0.2 and 0.5 SD depending on the outcome and child sex.

### Sensitivity analyses

First, we examined whether there was a linear association between maternal education and mental health outcomes by plotting trajectories separately for each category of maternal education (Supplementary Figs. 1 and 2). For most cohorts we observed a linear association, with the exception of DNBC (for both sexes and outcomes) and Gen‐R (both sexes for internalising). Second, we compared findings using disposable income as a complimentary indicator of SEP (Supplementary Figs. 3 and 4). Inequalities were generally smaller, especially for EDEN, MoBa & Rhea. Third, we repeated analyses in ALSPAC, GenR & the Raine study additionally adjusting for ethnicity (Supplementary Figs. 5 and 6). For Gen‐R adjusting for ethnicity slightly decreased estimates of inequality, whilst for ALSPAC & the Raine study estimates were unchanged. Finally, adjusting for maternal mental health did not markedly change estimates for any cohort (Supplementary Figs. 7 and 8).

## Discussion

In the largest study of its kind, we have used data from up to 149,604 children across eight birth cohorts to study how social inequalities in child mental health develop and change over time. For both internalising and externalising problems, we found a consistent pattern that children born into more deprived SECs had more problems than children born into less deprived SECs. Whilst patterns of change in inequalities as children aged varied between cohorts, for almost all cohorts inequalities remained at the oldest measurement age.

### Strengths and limitations

One of the major strengths of this study is its large geographical coverage including different regions of Europe and Australia, enabling us to examine social inequalities across twelve affluent countries with different cultural and social settings. We included individual level data on maternal education which was harmonised according to the International Standard Classification of Education [33]. We also included data on internalising and externalising problems spanning the whole of childhood (ages 2 ‐ 18 years).

There were however limitations. First, due to technical limitations with the DataSHIELD infrastructure multiple imputation was not available. We were therefore limited to using complete case analysis on exposure and covariate data. Second, participation in birth cohort studies is often associated with socio‐economic position thus these results may not be representative of the underlying population. Furthermore, all cohorts suffer attrition over time which is often associated with socio‐economic factors [41], however we partially mitigated this through our use of full‐information maximum likelihood estimation. Third, whilst fractional polynomial models are effective at accounting for complex non‐linear change, they are also prone to over‐fitting the data, especially for the cohorts with smaller sample sizes. Fourth, different questionnaires were used to measure internalising and externalising problems in different cohorts, therefore outcomes are not entirely comparable. For example, whilst the SDQ always incorporates attentional problems and hyperactivity in the externalising scale, in the CBCL attention problems only form part of the scale for children aged under five (GenR, MoBa). Additionally, almost all outcomes were measured by parent-report, which may be at risk of proxy-reporting bias. However, there is evidence that whilst agreement between self- and parent- reported SDQ scores is only moderate, both are good predictors of clinical diagnosis [42]. Mothers from different ethnic backgrounds may also respond differently (e.g. Gen-R had a high proportion of Non-Western mothers), however there is evidence that the CBCL performs well across different ethnic groups [43]. Fifth, because we calculated z‐scores within each cohort, a standard deviation change in internalising or externalising score will depend on the distribution within each cohort and will not have the same absolute magnitude. Sixth, longitudinal information on maternal education was not available thus we were unable to model how change in education level over time related to child mental health inequalities. Finally, we also lacked information in some cohorts for potential confounders.

### Interpretation of findings

We found evidence that from as young as age two, children born into more disadvantaged SECs had more internalising and externalising problems (GenR and MoBa), demonstrating that social inequalities in mental health problems are established very early in life. Where cohorts first measured mental health problems at older ages, inequalities were also present (ALSPAC, CHOP, DNBC, Eden & Raine, ages 4‐7). These results are largely consistent with previous research. For example, two studies using the UK Millenium Cohort Study reported that children born to more economically deprived families had higher levels of internalising and externalising problems as young as age 3 [10, 11]. Similarly, a study pooling survey data across seven EU countries (Austria, France, Germany, Netherlands, Spain, Switzerland, and the United Kingdom) found that lower maternal education and income were associated with lower psychological well‐being at ages 8‐11 [13]. We extend these findings by showing that these patterns are consistent across other European countries with inequalities present from a young age.

We found some exceptions to these patterns. For example, for boys in the Raine study we found little evidence of inequalities for either outcome. This is in contrast to a recent study using a national Australian cohort (Longitudinal Study of Australian Children), which found that children from lower income families had higher emotional problems at age five [16]. However, in our study disposable income was not available for the Raine study, so this difference could be explained by differences in measure of SEP as well as the different location of participants in the two studies (metropolitan Perth vs nationwide). In general, differences between cohorts may be attributable to the many demographic differences between the populations included (e.g. age of parents, ethnicity, years of data collection).

There are both causal and non‐causal interpretations of the association between lower SEP and higher internalising and externalising problems. Possible causal mechanisms include families of lower SEP being exposed to more traumatic events and stressors, greater financial stress which could lead to family disruption (e.g. conflict and separation), stresses associated with living in more disadvantaged neighbourhoods, or via biological pathways such as poorer nutrition or more frequent maternal smoking [44–47]. As outcomes were almost exclusively rated by parents, it is also possible that parental factors may have biased these ratings. For example, if families with lower SEP experience greater stress, they may rate their children as having worse problems inflating the true association. Conversely such effects could also work in the opposite direction, for example if families from lower SEP background attach greater stigma to mental health problems, they may rate their children as having fewer problems which would bias the true association towards null.

Our findings suggest that the pattern of inequalities as children age depends on country, sex and outcome. This is reflected in previous literature which has reported both increasing and decreasing inequalities. For example, two previous studies (which used partially overlapping data to that included here) found narrowing of inequalities in internalising and externalising between ages 7 to 11 (ALSPAC) and 2 to 9 (GenR) [3, 17]. A multi‐cohort study using an Australian and British cohort also found differences in emotional problems at age 5 which remained at age 14 [16]. Two previous studies have reported widening of inequalities. A study in the Netherlands found that social inequalities in teacher and self‐rated emotional and behavioural problems increased between ages 7 to 12 [14], whilst a UK study using the Millenium Birth Cohort found that chronic poverty was associated with an increase in internalising and externalising problems between ages 5‐7 [15]. However, the data could not show whether this early‐age widening would have continued at later ages, or whether it was part of a non‐linear trend. A strength of the present study was that we were able to model inequalities over a wide age range, which allowed us to observe non‐linear trends such as slight widening of inequalities at younger ages which then narrowed as children moved into adolescence (e.g. DNBC).

The decrease of inequalities observed in Denmark, Netherlands and United Kingdom could reflect a process of equalisation [48]. Assuming that at least some of the pathway from SECs to poor health is via the home environment (i.e. not entirely through neighbourhood factors), then as children age the impact of the home environment will lessen whilst outside influences increase [48]. External influences such as the school environment and mixing with children from other socio‐economic backgrounds could have an ameliorative effect on mental health, for example through decreased stigmatisation of mental health or through additional support provided by teachers. However, this decrease did not occur in all countries, and despite this reduction we found inequalities to persist at the oldest measurement wave. This is again consistent with previous findings, for example a UK study using data from the Millennium Cohort Study, which reported that chronic poverty was associated with 4 times risk of mental health problems at age 17 [49].

## Conclusions

Addressing inequalities in mental health over the lifecourse is a major public health challenge. In this study we show social inequalities in internalising and externalising problems across multiple (mostly European) countries. We observed differences from as young as two years, with children born to more deprivied SECs having higher levels of internalising and externalising problems. Furthermore, we extend previous studies by showing this pattern to be largely consistent across different countries. Whilst in some cohorts these differences decreased over childhood, inequalities largely remained at the oldest age of measurement. We thus show that social inequalities in mental health emerge early in life and persist into adolescence. Efforts to reduce inequalities in adolescent’s mental health problems should focus on reducing socio‐economic inequalities and to identifying and targeting potential mediators of this adverse effect that starts early in life.

## Supplementary Information

Supplementary Material 1.

## Data Availability

The data that support the findings of this study are available from the individual cohort studies included, but restrictions apply to the availability of these data, which were used under license for the current study, and so are not publicly available.

## References

[1] Reiss F. Socioeconomic inequalities and mental health problems in children andadolescents: a systematic review. Soc Sci Med. 2013;90:24–31. 23746605 10.1016/j.socscimed.2013.04.026

[2] Silva M, Loureiro A, Cardoso G. Social determinants of mental health: a review of the evidence. Eur J Psychiatry. 2016;30(4):259–92.

[3] Howe LD, Lawlor DA, Propper C. Trajectories of socioeconomic inequalities in health, behaviours and academic achievement across childhood andadolescence. J Epidemiol Community Health. 2013;67(4):358–64. 23322849 10.1136/jech-2012-201892 PMC3596772

[4] Hargrove TW. Intersecting social inequalities and body mass index trajectories from adolescence to early adulthood. J Health Soc Behav. 2018;59(1):56–73. 29300495 10.1177/0022146517746672 PMC6561119

[5] Howe LD, et al. Socioeconomic differences in childhood growth trajectories: at what age do height inequalities emerge? J Epidemiol Community Health. 2012;66(2):143–8. 20724285 10.1136/jech.2010.113068 PMC3245896

[6] Wijlaars LP, et al. Socioeconomic status and weight gain in early infancy. Int J Obes. 2011;35(7):963–70. 10.1038/ijo.2011.88 PMC3145137 21540830

[7] Bramsved R, et al. Parental education and family income affect birthweight, early longitudinal growth and body mass index development differently. Acta Paediatrica. 2018;107(11):1946–52. 29315777 10.1111/apa.14215

[8] McCrory C, et al. Socioeconomic differences in children’s growth trajectories from infancy to early adulthood: evidence from four European countries. J Epidemiol Community Health. 2017;71(10):981–9. 28798151 10.1136/jech-2016-208556

[9] Patel R, et al. Socioeconomic differences in childhood BMI trajectories in Belarus. Int J Obes. 2018;42(9):1651–60. 10.1038/s41366-018-0042-0 PMC6033313 29568106

[10] Kiernan KE, Huerta MC. Economic deprivation, maternal depression, parenting and children’s cognitive and emotional development in early childhood 1. Br J Sociol. 2008;59(4):783–806. 19035922 10.1111/j.1468-4446.2008.00219.x

[11] Kiernan KE, Mensah FK. Poverty, maternal depression, family status and children’s cognitive and behavioural development in early childhood: A longitudinal study. J Social Policy. 2009;38(4):569–88.

[12] A C Kalff et al. “Neighbourhood level and individual level SES effects on child problem behaviour: a multilevel analysis”. In: Journal of Epidemiology & Community Health 55.4 (2001), pp. 246–250. ISSN: 0143‐005X. 10.1136/jech.55.4.246. eprint: https://jech.bmj.com/content/55/4/246.full.pdf. URL: https://jech.bmj.com/content/55/4/246. 10.1136/jech.55.4.246 PMC1731860 11238579

[13] Ursula von Rueden et al. “Socioeconomic determinants of health related quality of life in childhood and adolescence: results from a European study”. In: Journal of Epidemiology &Community Health 60.2 (2006), pp. 130–135. ISSN: 0143‐005X. 10.1136/jech.2005.039792. eprint: https://jech.bmj.com/content/60/2/130.full.pdf. URL: https://jech.bmj.com/content/60/2/130. 10.1136/jech.2005.039792 PMC2566139 16415261

[14] Nil Horoz et al. “Children’s behavioral and emotional problems and peer relationships across elementary school: Associations with individual‐ and school‐level parental education”. In: Journal of School Psychology 93 (2022), pp. 119–137. ISSN: 0022‐4405. 10.1016/j.jsp.2022.06.005. URL: https://www.sciencedirect.com/science/article/pii/S0022440522000528. 10.1016/j.jsp.2022.06.005 35934447

[15] Flouri E, Midouhas E. School composition, family poverty and child behaviour. Soc Psychiatry Psychiatr Epidemiol. 2016;51:817–26. 27059661 10.1007/s00127-016-1206-7 PMC4889629

[16] Sonia Terhaag et al. “Sex, ethnic and socioeconomic inequalities and trajectories in child and adolescent mental health in Australia and the UK: findings from national prospective longitudinal studies”. In: J Child Psychol Psychiatry. 2021;62(10):1255–1267. 10.1111/jcpp.13410. URL: https://acamh.onlinelibrary.wiley.com/doi/abs/10.1111/jcpp.13410. 10.1111/jcpp.13410 33948953

[17] Houweling TA, et al. Trajectories of socioeconomic inequality in early child development: a cohort analysis. Int J Equity Health. 2002;21(1):1–10. 10.1186/s12939-022-01675-8 PMC9172194 35668449

[18] Elovainio M, et al. Socioeconomic status and the development of depressive symptoms from childhood to adulthood: a longitudinal analysis across 27 years of follow-up in the Young Finns study. Soc Sci Med. 2012;74(6):923–9. 22305468 10.1016/j.socscimed.2011.12.017

[19] Noonan K, Burns R, Violato M. Family income, maternal psychological distress and child socio-emotional behaviour: Longitudinal findings from the UK Millennium Cohort Study. SSM-population health. 2018;4:280–90. 29854912 10.1016/j.ssmph.2018.03.002 PMC5976845

[20] Jaddoe VWV, et al. The LifeCycle Project-EU Child Cohort Network: a federated analysis infrastructure and harmonized data of more than 250,000 children and parents. Eur J Epidemiol. 2020;35:709–24. 32705500 10.1007/s10654-020-00662-z PMC7387322

[21] Vergunst F, et al. Association of childhood externalizing, internalizing, and comorbid symptoms with long-term economic and social outcomes. JAMA Network Open. 2023;6(1):e2249568–e2249568. 36622675 10.1001/jamanetworkopen.2022.49568 PMC9856729

[22] Galobardes B, et al. Indicators of socioeconomic position (part 1). J Epidemiol Commun Health. 2006;60(1):7–12. 10.1136/jech.2004.023531 PMC2465546 16361448

[23] Boyd A, et al. Cohort profile: the ‘children of the 90s’—the index offspring of the Avon Longitudinal Study of Parents and Children. Int J Epidemiol. 2013;42(1):111–27. 22507743 10.1093/ije/dys064 PMC3600618

[24] Fraser A, et al. Cohort profile: the Avon Longitudinal Study of Parents and Children: ALSPAC mothers cohort. Int J Epidemiol. 2013;42(1):97–110. 22507742 10.1093/ije/dys066 PMC3600619

[25] Koletzko B, von Kries R, Closa R, Escribano J, Scaglioni S, Giovannini M, Beyer J, Demmelmair H, Gruszfeld D, Dobrzanska A, Sengier A, Langhendries JP, Rolland Cachera MF, Grote V, European Childhood Obesity Trial Study Group. Lower protein in infant formula is associated with lower weight up to age 2 y: a randomized clinical trial. Am J Clin Nutr. 2009;89(6):1836–45. 10.3945/ajcn.2008.27091. 19386747 10.3945/ajcn.2008.27091

[26] Olsen J, et al. The Danish National Birth Cohort-its background, structure and aim. Scandinavian J Public Health. 2001;29(4):300–7. 10.1177/14034948010290040201 11775787

[27] Heude B, et al. Cohort Profile: The EDEN mother-child cohort on the prenatal and early postnatal determinants of child health and development. Int J Epidemiol. 2016;45(2):353–63. 26283636 10.1093/ije/dyv151

[28] Jaddoe VWV, et al. The Generation R Study: design and cohort update 2012. Eur J Epidemiol. 2012;27:739–56. 23086283 10.1007/s10654-012-9735-1

[29] Magnus P, et al. Cohort profile: the Norwegian mother and child cohort study (MoBa)”. Int J Epidemiol. 2006;35(5):1146–50. 16926217 10.1093/ije/dyl170

[30] Newnham JP, et al. Effects of frequent ultrasound during pregnancy: a randomised controlled trial. Lancet. 1993;342(8876):887–91. 8105165 10.1016/0140-6736(93)91944-h

[31] Chatzi L, et al. Metabolic syndrome in early pregnancy and risk of preterm birth. Am J Epidemiol. 2009;170(7):829–36. 19713286 10.1093/aje/kwp211

[32] Nader JL, et al. Measures of Early-life Behavior and Later Psychopathology in the LifeCycle Project - EU Child Cohort Network: A Cohort Description. J Epidemiol. 2023;33(6):321–31. 10.2188/jea.JE20210241. 34776498 10.2188/jea.JE20210241 PMC10165218

[33] Wolf C, Hoffmeyer-Zlotnik JHP. Measuring Demographic and Socio-Economic Variables in Cross-National Research. In: Hoffmeyer-Zlotnik JHP, Wolf C, editors. Advances in Cross-National Comparison. Boston: Springer; 2003. 10.1007/978-1-4419-9186-7_1.

[34] Enrique Regidor. “Measures of health inequalities: part 2”. J Epidemiol Community Health. 2004;58(11):900–903. ISSN: 0143‐005X. 10.1136/jech.2004.023036. eprint: https://jech.bmj.com/content/58/11/900.full.pdf. URL: https://jech.bmj.com/content/58/11/900. 10.1136/jech.2004.023036 PMC1732621 15483304

[35] Goodman R. The Strengths and Difficulties Questionnaire: a research note. J Child Psychol Psychiatry. 1997;38(5):581–6. 9255702 10.1111/j.1469-7610.1997.tb01545.x

[36] Achenbach TM, Edelbrock C. Child behavior checklist. Burlington (Vt). 1991;7:371–92.

[37] Gaye A, et al. DataSHIELD: taking the analysis to the data, not the data to the analysis. Int J Epidemiol. 2014;43(6):1929–44. 25261970 10.1093/ije/dyu188 PMC4276062

[38] Tilling K, et al. Modelling childhood growth using fractional polynomials and linear splines. Ann NutrMetabol. 2014;65(2–3):129–38. 10.1159/000362695 PMC4264511 25413651

[39] Pizzi C, et al. Measuring child socio-economic position in birth cohort research: the development of a novel standardized household income indicator. Int J Environ Res Public Health. 2020;17(5):1700. 32150940 10.3390/ijerph17051700 PMC7084936

[40] Larsen R. “Missing Data Imputation versus Full Information Maximum Likelihood with Second‐Level Dependencies”. Structural Equation Modeling: A Multidisciplinary J. 2011;18(4):649–662. 10.1080/10705511.2011.607721.

[41] Howe LD, et al. “Loss to follow-up in cohort studies: bias in estimates of socioeconomic inequalities. Epidemiol (Cambridge, Mass). 2013;24(1):1. 10.1097/EDE.0b013e31827623b1 PMC5102324 23211345

[42] Andreas B, et al. Evaluation of the self-reported SDQ in a clinical setting: do self-reports tell us more than ratings by adult informants. Eur Child Adolesc Psychiatry. 2004;13:ii17–24. 15243782 10.1007/s00787-004-2004-4

[43] Deborah G, et al. The equivalence of the Child Behavior Checklist/1 1/2–5 across parent race/ethnicity, income level, and language. Psychol Assess. 2006;18(3):313. 16953734 10.1037/1040-3590.18.3.313

[44] Viviane S Straatmann et al. “How do early‐life factors explain social inequalities in adolescent mental health? Findings from the UK Millennium Cohort Study”. In: J Epidemiol Community Health. 2019;73(11):1049–1060. ISSN: 0143‐005X. 10.1136/jech-2019-212367. eprint: https://jech.bmj.com/content/73/11/1049.full.pdf. URL: https://jech.bmj.com/content/73/11/1049. 10.1136/jech-2019-212367 PMC6877708 31492761

[45] Mikhail JN, et al. The social determinants of trauma: a trauma disparities scoping review and framework. J Trauma Nursing JTN. 2018;25(5):266–81. 10.1097/JTN.0000000000000388 30216255

[46] McLaughlin KA, et al. Childhood socio-economic status and the onset, persistence, and severity of DSM-IV mental disorders in a US national sample”. Soc Sci Med. 2001;73(7):1088–96. 10.1016/j.socscimed.2011.06.011 PMC3191493 21820781

[47] Eric T, Lai C, et al. “Pathways to inequalities in child mental health: a causal mediation analysis of evidence from two national birth cohorts”. Lancet. 2019;394. Public Health Science 2019, S3. ISSN: 0140‐6736. 10.1016/S0140-6736(19)32800-4. URL: https://www.sciencedirect.com/science/article/pii/S0140673619328004.

[48] West P. Health inequalities in the early years: is there equalisation in youth? Soc Sci Med. 1997;44(6):833–58. 9080566 10.1016/s0277-9536(96)00188-8

[49] Nicholas Kofi Adjei et al. “Impact of Parental Mental Health and Poverty on the Health of the Next Generation: A Multi‐Trajectory Analysis Using the UK Millennium Cohort Study”. In: Journal of Adolescent Health (2023). 10.1016/j.jadohealth.2023.07.029 37831048

