# Supplementary material for: Social inequalities in child mental health trajectories: a longitudinal study using birth cohort data 12 countries
Source: BMC Public Health. 2024 Oct 22;24:2930. doi: 10.1186/s12889-024-20291-5 (PMC11515779; doi:10.1186/s12889-024-20291-5)
Supplement: Supplementary file 1 — Supplementary Material 1. [file 12889_2024_20291_MOESM1_ESM.docx]

**Supplementary Text 1: Study specific information**

# ALSPAC

Pregnant women resident in Avon, UK with expected dates of delivery 1st April 1991 to 31st December 1992 were invited to take part in the study. The initial number of pregnancies enrolled is 14,541 (for these at least one questionnaire has been returned or a “Children in Focus” clinic had been attended by 19/07/99). Of these initial pregnancies, there was a total of 14,676 foetuses, resulting in 14,062 live births and 13,988 children who were alive at 1 year of age.

When the oldest children were approximately 7 years of age, an attempt was made to bolster the initial sample with eligible cases who had failed to join the study originally. As a result, when considering variables collected from the age of seven onwards (and potentially abstracted from obstetric notes) there are data available for more than the 14,541 pregnancies mentioned above. The number of new pregnancies not in the initial sample (known as Phase I enrolment) that are currently represented in the released data and reflecting enrolment status at the age of 24 is 906, resulting in an additional 913 children being enrolled (456, 262 and 195 recruited during Phases II, III and IV respectively). The phases of enrolment are described in more detail in the cohort profile paper and its update. The total sample size for analyses using any data collected after the age of seven is therefore 15,447 pregnancies, resulting in 15,658 foetuses. Of these 14,901 were alive at 1 year of age.

A 10% sample of the ALSPAC cohort, known as the Children in Focus (CiF) group, attended clinics at the University of Bristol at various time intervals between 4 to 61 months of age. The CiF group were chosen at random from the last 6 months of ALSPAC births (1432 families attended at least one clinic). Excluded were those mothers who had moved out of the area or were lost to follow‐up, and those partaking in another study of infant development in Avon.

Full details of the cohort can be provided in the cohort profiles [1, 2]. Please note that the study website contains details of all the data that is available through a fully searchable data dictionary and variable search tool: http://www.bristol.ac.uk/alspac/researchers/our‐data/

The UK Medical Research Council and Wellcome (Grant ref: 217065/Z/19/Z) and the University of Bristol provide core support for ALSPAC. This publication is the work of the authors and Tim Cadman and Deborah Lawlor will serve as guarantors for the contents of this paper. A comprehensive list of grants funding is available on the ALSPAC website (http://www.bristol.ac.uk/alspac/external/documents/grant‐acknowledgements.pdf); This research was specifically funded by H2020 LifeCycle project Grant Agreement No. 733206).

Ethical approval for the study was obtained from the ALSPAC Ethics and Law Committee and the Local Research Ethics Committees. Informed consent for the use of data collected via questionnaires and clinics was obtained from participants following the recommendations of the ALSPAC Ethics and Law Committee at the time.

We are extremely grateful to all of the families who took part in ALSPAC, the midwives for their help in recruiting them, and the whole ALSPAC team, which includes interviewers, computer and laboratory technicians, clerical workers, research scientists, volunteers, managers, receptionists and nurses.

# CHOP

The CHOP study has been carried out with partial financial support from the Commission of the European Community, specific RTD Programme ”Quality of Life and Management of

Living Resources”, within the Fifth Framework Program (research grants no. QLRT‐2001‐00389 and QLK1‐CT‐200230582), the Sixth Framework Program (contract no. 007036), and

Seventh Framework Programme (EarlyNutrition; grant agreement no. 289346), the EU H2020 project LIFECYCLE under grant no. 733206 and the European Research Council Advanced Grant META‐GROWTH (ERC‐2012‐AdG – no.322605) and with financial support from Polish Ministry of Science and Higher Education (2571/7.PR/2012/2). This manuscript does not necessarily reflect the views of the Commission and in no way anticipates the future policy in this area. No funding bodies had any role in the study design, data collection and analysis. The study was approved by the ethics committees of all study centers. Written informed parental consent was obtained for each infant.

The authors would particularly like to thank all the cohort participants for their generous collaboration. Furthermore, thanks to all persons who designed and conducted the study, entered the data, and participated in the data analysis and who are represented by the European Childhood Obesity Trial Study Group participants: B Koletzko, V Grote, M Totzauer, K Gürlich, P Schwarzfischer, N Aumüller, V Luque, M Zaragoza‐Jordana, N Ferré, J Escribano, R Closa‐Monasterolo, A Xhonneux, JP Langhendries, E Verduci, E Riva, D Gruszfeld.

# DNBC

The Danish National Birth Cohort was established with a significant grant from the Danish National Research Foundation. Additional support was obtained from the Danish Regional Committees, the Pharmacy Foundation, the Egmont Foundation, the March of Dimes Birth Defects Foundation, the Health Foundation and other minor grants. The DNBC Biobank has been supported by the Novo Nordisk Foundation and the Lundbeck Foundation. Follow‐up of mothers and children have been supported by the Danish Medical Research Council (SSVF 0646, 271‐08‐0839/06‐066023, O602‐01042B, 0602‐02738B), the Lundbeck Foundation (195/04, R100‐A9193), The Innovation Fund Denmark 0603‐00294B (09‐067124), the Nordea Foundation (02‐2013‐2014), Aarhus Ideas (AU R9‐A959‐13‐S804), University of Copenhagen Strategic Grant (IFSV 2012), and the Danish Council for Independent Research (DFF – 4183‐00594 and DFF ‐ 4183‐00152). AP is funded by a Lundbeck Foundation fellowship (R264‐2017‐3099).

The DNBC complies with the Declaration of Helsinki and was approved by the Danish National Committee on Biomedical Research Ethics. Informed consent was obtained from participants upon enrolment.

The authors would like to thank the participants, the first Principal Investigator of DNBC Prof. Jørn Olsen, the scientific managerial team, and DNBC secretariat for being, establishing, developing and consolidating the Danish National Birth Cohort.

# EDEN

The EDEN study was supported by Foundation for medical research (FRM), National Agency for Research (ANR), National Institute for Research in Public health (IRESP: TGIR cohorte santé 2008 program), French Ministry of Health (DGS), French Ministry of Research, INSERM Bone and Joint Diseases National Research (PRO‐A) and Human Nutrition National Research Programs, Paris‐Sud University, Nestlé, French National Institute for Population Health Surveillance (InVS), French National Institute for Health Education (INPES), the

European Union FP7 programmes (FP7/2007‐2013, HELIX, ESCAPE, ENRIECO, Medall projects), Diabetes National Research Program (through a collaboration with the French Association of Diabetic Patients (AFD)), French Agency for Environmental Health Safety (now ANSES), Mutuelle Générale de l’Education Nationale a complementary health insurance (MGEN), French national agency for food security, French speaking association for the study of diabetes and metabolism (ALFEDIAM).

The study received approval from the ethics committee (CCPPRB) of Kremlin Bicêtre on 12 December 2002 and from CNIL (Commission Nationale Informatique et Liberté), the French data privacy institution. Women gave written informed consent for themselves and their child. Fathers gave written informed consent for themselves.

The authors thank the cohort participants and the EDEN mother‐child study group, whose members are: I. Annesi‐Maesano, J.Y. Bernard, J. Botton, M.A. Charles, P.

Dargent‐Molina, B. de Lauzon‐Guillain, P. Ducimetière, M. de Agostini, B. Foliguet, A. Forhan, X. Fritel, A. Germa, V. Goua, R. Hankard, B. Heude, M. Kaminski, B. Larroque†, N. Lelong, J. Lepeule, G. Magnin, L. Marchand, C. Nabet, F Pierre, R. Slama, M.J. Saurel‐Cubizolles, M. Schweitzer, O. Thiebaugeorges.

## GenR

The general design of the Generation R Study is made possible by financial support from the Erasmus MC, University Medical Center, Rotterdam, Erasmus University Rotterdam, Netherlands Organization for Health Research and Development (ZonMw), Netherlands Organisation for Scientific Research (NWO), Ministry of Health, Welfare and Sport and Ministry of Youth and Families. This project received funding from the European Union’s Horizon 2020 research and innovation programme (LIFECYCLE, grant agreement No 733206, 2016; EUCAN‐Connect grant agreement No 824989; ATHLETE, grant agreement No 874583). VJ received funding from a Consolidator Grant from the European Research Council (ERC‐2014‐CoG‐648916). The study sponsors had no role in the study design, data analysis, interpretation of data, or writing of this report.

The general design, all research aims and the specific measurements in the Generation R Study have been approved by the Medical Ethical Committee of the Erasmus Medical Center, Rotterdam. New measurements will only be embedded in the study after approval of the Medical Ethical Committee. Participants are asked for their written informed consent for the four consecutive phases of the study (prenatally, birth to 4 years, 4–12 years, and from 12 years onwards). At the start of each phase, mothers and their partners receive written and oral information about the study. Even with consent of the parents, when the child is not willing to participate actively, no measurements are performed. From the age of 12 years, children are asked for written informed consent.

The authors gratefully acknowledge the contribution of participants, research collaborators, general practitioners, hospitals, midwives, and pharmacies in Rotterdam.

## MoBa

The Norwegian Mother, Father and Child Cohort Study is supported by the Norwegian Ministry of Health and Care Services and the Ministry of Education and Research. The establishment and data collection in MoBa was previously based on a license from the Norwegian Data protection agency and approval from The Regional Committee for Medical Research Ethics, and it is now based on regulations related to the Norwegian Health Registry Act. MoBa is conducted according to the guidelines laid down in the declaration of Helsinki, and written informed consent was obtained from all participants. A detailed protocol of the study including the consent can be found elsewhere

(http://www.fhi.no/morogbarn). JRH is supported, in part, by The Research Council of Norway through its Centres of Excellence funding scheme, project nr. 262700.

The authors are grateful to all the participating families in Norway who take part in this on‐going cohort study.

## Raine

We would like to acknowledge the Raine Study participants and their families for their ongoing participation in the study and the Raine Study team for study co‐ordination and data collection. We also thank the NHMRC for their long term contribution to funding the study over the last 30 years. The core management of the Raine Study is funded by The University of Western Australia, Curtin University, Telethon Kids Institute, Women and Infants Research Foundation, Edith Cowan University, Murdoch University, The University of Notre Dame Australia and the Raine Medical Research Foundation.

The Raine Study has been funded by program and project grants from the Australian National Health and Medical Research Council (NHMRC), the Commonwealth Scientific and Industrial Research Organisation, Healthway and the Lions Eye Institute in Western Australia. The Raine study Gen2‐17 year follow‐up was funded by the NHMRC Program Grant (Stanley et al, ID 353514). The Raine study participation in LIFECYCLE was funded by a grant from the National Health and Medical Research Council, Australia (GNT114285). The University of Western Australia (UWA), Curtin University, the Raine Medical Research Foundation, the Telethon Kids Institute, the Women’s and Infant’s Research Foundation (KEMH), Murdoch University, The University of Notre Dame Australia and Edith Cowan University provide funding for the Core Management of the Raine Study. RCH was supported by NHMRC fellowship (Grant Number 1053384). Ethics approval was obtained from the Human Ethics Committees at King Edward Memorial Hospital, Princess Margaret Hospital, The University of Western Australia and Curtin University. All participants and guardians provided written consent.

## Rhea

The Mother‐Child Cohort Study (“Rhea”) was financially supported by European projects (EU FP6‐2003‐Food‐3‐NewGeneris, EU FP6. STREP Hiwate, EU FP7 NV.2007.1.2.2.2. Project

No 211250 Escape, EU FP7‐2008‐ENV‐1.2.1.4 Envirogenomarkers, EU FP7‐HEALTH‐2009‐ single stage CHICOS, EU FP7 ENV.2008.1.2.1.6. Proposal No 226285 ENRIECO, EU‐ FP7HEALTH‐2012 Proposal No 308333 HELIX, and EU H2020 projects LIFECYCLE grant agreement no. 733206, ATHLETE, grant agreement No 874583) and the Greek Ministry of Health (Program of Prevention of obesity and neurodevelopmental disorders in preschool children, in Heraklion district, Crete, Greece: 2011‐2014; “Rhea Plus”: Primary Prevention Program of Environmental Risk Factors for Reproductive Health, and Child Health: 2012‐15).

The study was approved by the corresponding ethical committees. All participants provided written, informed consent.

The authors would particularly like to thank all the cohort participants for their generous collaboration.

## **Supplementary Text 2: Model equations**

The general form of the linear effects regression model is:

*Y_ij_* = (*β*0+*µ*_0_*_j_*)+(*β*1)(*mated_j_*)+(*β*2)(*age_ij_*)*^p^*^1^ +(*β*3)(*age_ij_*)*^p^*^2^ +(*β*4)(*mated_j_*)(*age_ij_*)*^p^*^1^ +(*β*5)(*mated_j_*)(*age_ij_*)*^p^*^2^ +*covariates_j_* +*e_ij_*

Where Y is the outcome for person j at time i, age is the age in years at measurement, mated is rank score for (maternal education or disposable income), β0 = average value of outcome at first measurement occasion, β1 = average change in outcome between hypothetical case of 100% of sample having lowest level of maternal education vs highest (SII), β2 & β3 = average change in outcome per year age (transformed to the power of p1 (child age ^‐2^) and p2 (child age ^‐1^), which represent the powers providing best model fit) and β4 & β5 = average change in SII per year age (age transformed as described), µ = random effect and e = residual. For each outcome, we compared models using different combinations of fractional polynomials (‐2, ‐1, ‐0.5, log, 0.5, 2, 3) and chose the best fitting model.

Supplementary Table 1: Comparison between included and excluded participants: ALSPAC, CHOP, DNBC & EDEN

**ALSPAC**

**CHOP**

**DNBC**

**EDEN**

|  |  | Included (N = 9707) | Excluded (N = 5938) | Included (N = 662) | Excluded (N = 1016) | Included (N = 55163) | Excluded (N = 41662) | Included (N = 1426) | Excluded (N = 576) |
| --- | --- | --- | --- | --- | --- | --- | --- | --- | --- |
| Maternal Education | High | 1420 (14.6) | 625 (10.5) | 206 (31.1) | 236 (23.2) | 28018 (49.4) | 3152 (7.87) | 840 (58.9) | 296 (51.4) |
|  | Medium | 6733 (69.4) | 3159 (53.2) | 338 (51.1) | 519 (51.1) | 21071 (37.1) | 3550 (8.86) | 519 (36.4) | 209 (36.3) |
|  | Low | 1554 (16) | 977 (16.4) | 118 (17.8) | 256 (25.2) | 7666 (13.5) | 1680 (4.19) | 67 (4.7) | 45 (7.81) |
|  | NA | 0 (0) | 1177 (19.8) | 0 (0) | 5 (0.49) | 0 (0) | 31688 (79.1) | 0 (0) | 26 (4.51) |
| Disposable Income | 1st quintile | 1418 (14.6) | 802 (13.5) | 79 (11.9) | 190 (18.7) | 9362 (16.5) | 7006 (17.5) | 191 (13.4) | 103 (17.9) |
|  | 2nd quintile | 1702 (17.5) | 857 (14.4) | 102 (15.4) | 186 (18.3) | 10098 (17.8) | 6372 (15.9) | 271 (19) | 113 (19.6) |
|  | 3rd quintile | 1845 (19) | 812 (13.7) | 133 (20.1) | 167 (16.4) | 10233 (18) | 6137 (15.3) | 301 (21.1) | 107 (18.6) |
|  | 4th quintile | 1857 (19.1) | 813 (13.7) | 157 (23.7) | 209 (20.6) | 10240 (18) | 6177 (15.4) | 295 (20.7) | 98 (17) |
|  | 5th quintile | 1879 (19.4) | 827 (13.9) | 160 (24.2) | 171 (16.8) | 10315 (18.2) | 6102 (15.2) | 320 (22.4) | 98 (17) |
|  | NA | 1006 (10.4) | 1827 (30.8) | 31 (4.68) | 93 (9.15) | 6507 (11.5) | 8276 (20.6) | 48 (3.37) | 57 (9.9) |
| Child sex | Male | 4990 (51.4) | 2909 (49) | 313 (47.3) | 517 (50.9) | 28244 (49.8) | 21400 (53.4) | 743 (52.1) | 300 (52.1) |
|  | Female | 4717 (48.6) | 2826 (47.6) | 349 (52.7) | 499 (49.1) | 28511 (50.2) | 18670 (46.6) | 683 (47.9) | 248 (43.1) |
|  | NA | 0 (0) | 203 (3.42) | 0 (0) | 0 (0) | 0 (0) | 0 (0) | 0 (0) | 28 (4.86) |
| Maternal age at birth | Mean ± SD | 28.8 ± 4.62 | 28.4 ± 4.72 | 30.9 ± 4.56 | 29.7 ± 5.1 | 30.1 ± 4.21 | 29.7 ± 4.46 | 30 ± 4.74 | 29.3 ± 5.02 |
|  | NA | 0 (0) | 1618 (27.2) | 0 (0) | 6 (0.59) | 0 (0) | 3 (0.01) | 0 (0) | 27 (4.69) |
| Maternal ethnicity | Western | 9464 (97.5) | 4604 (77.5) | 649 (98) | 962 (94.7) | 0 (0) | 0 (0) | 1246 (87.4) | 472 (81.9) |
|  | Non‐western | 129 (1.33) | 79 (1.33) | 13 (1.96) | 52 (5.12) | 0 (0) | 0 (0) | 6 (0.42) | 27 (4.69) |
|  | NA | 114 (1.17) | 1255 (21.1) | 0 (0) | 2 (0.2) | 56755 (100) | 40070 (100) | 174 (12.2) | 77 (13.4) |
| Maternal mental health | No | 8305 (85.6) | 4115 (69.3) | 0 (0) | 0 (0) | 50774 (89.5) | 32794 (81.8) | 1357 (95.2) | 521 (90.4) |
|  | Yes | 1023 (10.5) | 557 (9.38) | 0 (0) | 0 (0) | 3903 (6.88) | 3159 (7.88) | 69 (4.84) | 33 (5.73) |
|  | NA | 379 (3.9) | 1266 (21.3) | 662 (100) | 1016 (100) | 2078 (3.66) | 4117 (10.3) | 0 (0) | 22 (3.82) |

Note: Included participants comprises participants with complete data on maternal education, child sex, maternal age at birth and at least one measurement of either internalising or externalising at one time point. Excluded participants are participants from the original sample who did not meet these inclusion criteria.

Supplementary Table 2: Comparison between included and excluded participants: Gen‐R, Moba, The Raine Study & Rhea

**GenR**

**MoBa**

**The Raine study**

**Rhea**

|  |  | Included (N = 6726) | Excluded (N = 3175) | Included (N = 73042) | Excluded (N = 32269) | Included (N = 2294) | Excluded (N = 574) | Included (N = 584) | Excluded (N = 874) |
| --- | --- | --- | --- | --- | --- | --- | --- | --- | --- |
| Maternal Education | High | 3328 (49.5) | 385 (12.1) | 49404 (67.6) | 19160 (59.4) | 470 (20.5) | 45 (7.84) | 204 (34.9) | 178 (20.4) |
|  | Medium | 2863 (42.6) | 1117 (35.2) | 22250 (30.5) | 9911 (30.7) | 627 (27.3) | 67 (11.7) | 299 (51.2) | 384 (43.9) |
|  | Low | 535 (7.95) | 433 (13.6) | 1388 (1.9) | 852 (2.64) | 1197 (52.2) | 251 (43.7) | 81 (13.9) | 207 (23.7) |
|  | NA | 0 (0) | 1240 (39.1) | 0 (0) | 2346 (7.27) | 0 (0) | 211 (36.8) | 0 (0) | 105 (12) |
| Disposable Income | 1st quintile | 853 (12.7) | 429 (13.5) | 12553 (17.2) | 5577 (17.3) | 0 (0) | 0 (0) | 100 (17.1) | 130 (14.9) |
|  | 2nd quintile | 1030 (15.3) | 259 (8.16) | 12541 (17.2) | 5553 (17.2) | 0 (0) | 0 (0) | 103 (17.6) | 127 (14.5) |
|  | 3rd quintile | 1177 (17.5) | 114 (3.59) | 12690 (17.4) | 5406 (16.8) | 0 (0) | 0 (0) | 93 (15.9) | 138 (15.8) |
|  | 4th quintile | 1207 (18) | 84 (2.65) | 12482 (17.1) | 5624 (17.4) | 0 (0) | 0 (0) | 105 (18) | 125 (14.3) |
|  | 5th quintile | 1247 (18.5) | 46 (1.45) | 12471 (17.1) | 5547 (17.2) | 0 (0) | 0 (0) | 54 (9.25) | 176 (20.1) |
|  | NA | 1212 (18) | 2243 (70.6) | 10305 (14.1) | 4562 (14.1) | 2294 (100) | 574 (100) | 129 (22.1) | 178 (20.4) |
| Child sex | Male | 3386 (50.3) | 1552 (48.9) | 37400 (51.2) | 16456 (51) | 1177 (51.3) | 277 (48.3) | 327 (56) | 404 (46.2) |
|  | Female | 3340 (49.7) | 1468 (46.2) | 35642 (48.8) | 15742 (48.8) | 1117 (48.7) | 297 (51.7) | 257 (44) | 470 (53.8) |
|  | NA | 0 (0) | 155 (4.88) | 0 (0) | 71 (0.22) | 0 (0) | 0 (0) | 0 (0) | 0 (0) |
| Maternal age at birth | Mean ± SD | 31.2 ± 4.93 | 28.5 ± 5.77 | 30.4 ± 4.42 | 30.2 ± 4.58 | 28 ± 5.82 | 25.7 ± 6.08 | 30 ± 4.77 | 28.7 ± 5.2 |
|  | NA | 0 (0) | 156 (4.91) | 0 (0) | 55 (0.17) | 0 (0) | 77 (13.4) | 0 (0) | 50 (5.72) |
| Maternal ethnicity | Western | 4285 (63.7) | 755 (23.8) | 0 (0) | 0 (0) | 2059 (89.8) | 414 (72.1) | 575 (98.5) | 747 (85.5) |
|  | Non‐western | 2435 (36.2) | 1676 (52.8) | 0 (0) | 0 (0) | 235 (10.2) | 96 (16.7) | 3 (0.51) | 6 (0.69) |
|  | NA | 6 (0.09) | 744 (23.4) | 73042 (100) | 32269 (100) | 0 (0) | 64 (11.2) | 6 (1.03) | 121 (13.8) |
| Maternal mental health | No | 3104 (46.2) | 1472 (46.4) | 65873 (90.2) | 29108 (90.2) | 0 (0) | 0 (0) | 511 (87.5) | 772 (88.3) |
|  | Yes | 1340 (19.9) | 618 (19.5) | 5594 (7.66) | 2451 (7.6) | 0 (0) | 0 (0) | 14 (2.4) | 17 (1.95) |
|  | NA | 2282 (33.9) | 1085 (34.2) | 1575 (2.16) | 710 (2.2) | 2294 (100) | 574 (100) | 59 (10.1) | 85 (9.73) |

Note: Included participants comprises participants with complete data on maternal education, child sex, maternal age at birth and at least one measurement of either internalising or externalising at one time point. Excluded participants are participants from the original sample who did not meet these inclusion criteria.

Supplementary Table 3: Predicted Slope Index of Inequality for internalising symptoms in females

| Cohort | Age 2 | Age 3 | Age 4 | Age 5 | Age 6 | Age 7 | Age 8 | Age 10 | Age 12 | Age 15 | Age 18 |
| --- | --- | --- | --- | --- | --- | --- | --- | --- | --- | --- | --- |
| ALSPAC (UK) | _ | _ | 0.4 (0.2,  0.5) | 0.2 (0, 0.3) | 0.1 (0, 0.2) | 0.1 (0, 0.2) | 0.1 (0, 0.2) | 0.1 (0, 0.2) | 0.2 (0.1,  0.3) | 0.2 (0.1,  0.3) | _ |
| CHOP  (Multiple*) | _ | _ | _ | _ | 0.4 (0, 0.8) | 0.4 (0.1,  0.8) | 0.5 (0.1,  0.8) | 0.5 (0.1,  0.9) | _ | _ | _ |
| DNBC  (Denmark) | _ | _ | _ | _ | _ | 0.4 (0.3,  0.4) | 0.4 (0.4,  0.5) | 0.4 (0.4,  0.5) | 0.4 (0.4,  0.5) | 0.4 (0.3,  0.4) | 0.3 (0.3,  0.4) |
| EDEN  (France) | _ | 0.1 (0, 0.3) | 0.1 (‐0.1,  0.3) | 0.1 (‐0.1,  0.2) | 0 (‐0.1, 0.2) | 0 (‐0.1, 0.2) | 0 (‐0.1, 0.2) | _ | _ | _ | _ |
| GenR  (Netherlands) | 0.7 (0.5,  0.8) | _ | 0.5 (0.4,  0.6) | 0.4 (0.3,  0.6) | 0.4 (0.3,  0.5) | 0.4 (0.2,  0.5) | 0.3 (0.2,  0.5) | _ | _ | _ | _ |
| MoBa  (Norway) | 0.3 (0.2,  0.4) | _ | 0.2 (0.2,  0.3) | 0.2 (0.2,  0.3) | _ | _ | _ | _ | _ | _ | _ |
| The Raine study  (Australia) | _ | _ | 0.2 (‐0.1,  0.6) | 0.2 (‐0.1,  0.5) | 0.2 (‐0.1,  0.4) | 0.2 (0, 0.4) | 0.1 (‐0.1,  0.3) | 0.1 (‐0.1,  0.3) | _ | _ | _ |
| RHEA  (Greece) | _ | _ | _ | _ | 0.6 (0.1,  1.1) | 0.6 (0.2,  1.1) | 0.7 (0.3,  1.1) | 0.8 (0.3,  1.3) | _ | _ | _ |

Note: Model adjusted for maternal age birth. Values represent model‐predicted values. Predicted values shown from the youngest to oldest age of measurement.

Supplementary Table 4: Predicted Slope Index of Inequality for internalising symptoms in males

| Cohort | Age 2 | Age 3 | Age 4 | Age 5 | Age 6 | Age 7 | Age 8 | Age 10 | Age 12 | Age 15 | Age 18 |
| --- | --- | --- | --- | --- | --- | --- | --- | --- | --- | --- | --- |
| ALSPAC (UK) | _ | _ | 0.3 (0.2,  0.4) | 0.3 (0.1,  0.4) | 0.2 (0.1,  0.4) | 0.2 (0.1,  0.3) | 0.2 (0.1,  0.3) | 0.2 (0.1,  0.3) | 0.2 (0.1,  0.3) | 0.2 (0.1,  0.3) | _ |
| CHOP  (Multiple*) | _ | _ | _ | _ | 0.6 (0.1, 1) | 0.5 (0.1,  0.9) | 0.5 (0.1,  0.9) | 0.5 (0, 0.9) | _ | _ | _ |
| DNBC  (Denmark) | _ | _ | _ | _ | _ | 0.4 (0.3,  0.4) | 0.4 (0.3,  0.4) | 0.4 (0.3,  0.4) | 0.3 (0.3,  0.4) | 0.3 (0.2,  0.3) | 0.2 (0.2,  0.3) |
| EDEN  (France) | _ | 0.3 (0.1,  0.4) | 0.2 (0, 0.4) | 0.2 (0, 0.3) | 0.2 (0.1,  0.3) | 0.2 (0.1,  0.3) | 0.2 (0.1,  0.4) | _ | _ | _ | _ |
| GenR  (Netherlands) | 0.7 (0.5,  0.9) | _ | 0.5 (0.4,  0.7) | 0.5 (0.3,  0.6) | 0.4 (0.3,  0.5) | 0.3 (0.2,  0.5) | 0.3 (0.1,  0.4) | _ | _ | _ | _ |
| MoBa  (Norway) | 0.4 (0.3,  0.4) | _ | 0.3 (0.2,  0.3) | 0.3 (0.2,  0.4) | _ | _ | _ | _ | _ | _ | _ |
| The Raine study  (Australia) | _ | _ | ‐0.3 (‐0.6, 0.1) | ‐0.2 (‐0.5, 0.1) | ‐0.1 (‐0.3, 0.1) | 0 (‐0.2, 0.2) | 0.1 (‐0.1,  0.2) | 0.2 (‐0.1,  0.4) | _ | _ | _ |
| RHEA  (Greece) | _ | _ | _ | _ | 0.2 (‐0.3,  0.7) | 0.2 (‐0.3,  0.7) | 0.2 (‐0.3,  0.6) | 0.2 (‐0.3,  0.7) | _ | _ | _ |

Note: Model adjusted for maternal age birth. Values represent model‐predicted values. Predicted values shown from the youngest to oldest age of measurement.

Supplementary Table 5: Predicted Slope Index of Inequality for externalising symptoms in females

| Cohort | Age 2 | Age 3 | Age 4 | Age 5 | Age 6 | Age 7 | Age 8 | Age 10 | Age 12 | Age 15 | Age 18 |
| --- | --- | --- | --- | --- | --- | --- | --- | --- | --- | --- | --- |
| ALSPAC (UK) | _ | _ | 0.6 (0.5,  0.7) | 0.5 (0.4,  0.6) | 0.4 (0.3,  0.5) | 0.4 (0.3,  0.5) | 0.4 (0.3,  0.5) | 0.4 (0.3,  0.5) | 0.4 (0.3,  0.5) | 0.4 (0.3,  0.5) | _ |
| CHOP  (Multiple*) | _ | _ | _ | _ | 0.6 (0.2, 1) | 0.6 (0.2, 1) | 0.6 (0.3, 1) | 0.7 (0.3,  1.1) | _ | _ | _ |
| DNBC  (Denmark) | _ | _ | _ | _ | _ | 0.5 (0.5,  0.6) | 0.6 (0.5,  0.6) | 0.6 (0.5,  0.6) | 0.6 (0.5,  0.6) | 0.5 (0.5,  0.6) | 0.5 (0.4,  0.5) |
| EDEN  (France) | _ | 0.3 (0.2,  0.4) | 0.2 (0, 0.3) | 0.2 (0, 0.3) | 0.2 (0.1,  0.4) | 0.3 (0.2,  0.4) | 0.4 (0.2,  0.5) | _ | _ | _ | _ |
| GenR  (Netherlands) | 0.4 (0.2,  0.6) | _ | 0.4 (0.3,  0.5) | 0.4 (0.2,  0.5) | 0.3 (0.2,  0.5) | 0.3 (0.2,  0.4) | 0.3 (0.1,  0.4) | _ | _ | _ | _ |
| MoBa  (Norway) | 0.3 (0.3,  0.4) | _ | 0.4 (0.3,  0.4) | 0.4 (0.3,  0.4) | _ | _ | _ | _ | _ | _ | _ |
| The Raine study  (Australia) | _ | _ | 0.3 (0, 0.6) | 0.3 (0, 0.5) | 0.3 (0, 0.5) | 0.3 (0.1,  0.5) | 0.3 (0, 0.5) | 0.2 (0, 0.5) | _ | _ | _ |
| RHEA  (Greece) | _ | _ | _ | _ | 0.6 (0.1,  1.1) | 0.6 (0.2,  1.1) | 0.7 (0.3,  1.1) | 0.8 (0.3,  1.3) | _ | _ | _ |

Note: Model adjusted for maternal age birth. Values represent model‐predicted values. Predicted values shown from the youngest to oldest age of measurement.

Supplementary Table 6: Predicted Slope Index of Inequality for externalising symptoms in males

| Cohort | Age 2 | Age 3 | Age 4 | Age 5 | Age 6 | Age 7 | Age 8 | Age 10 | Age 12 | Age 15 | Age 18 |
| --- | --- | --- | --- | --- | --- | --- | --- | --- | --- | --- | --- |
| ALSPAC (UK) | _ | _ | 0.5 (0.4,  0.6) | 0.4 (0.3,  0.5) | 0.3 (0.2,  0.5) | 0.3 (0.2,  0.4) | 0.3 (0.2,  0.4) | 0.3 (0.2,  0.4) | 0.3 (0.2,  0.4) | 0.3 (0.1,  0.4) | _ |
| CHOP  (Multiple*) | _ | _ | _ | _ | 0.7 (0.3,  1.2) | 0.7 (0.3,  1.1) | 0.6 (0.2, 1) | 0.5 (0.1, 1) | _ | _ | _ |
| DNBC  (Denmark) | _ | _ | _ | _ | _ | 0.5 (0.5,  0.6) | 0.6 (0.5,  0.6) | 0.6 (0.5,  0.6) | 0.5 (0.5,  0.6) | 0.4 (0.4,  0.5) | 0.3 (0.3,  0.4) |
| EDEN  (France) | _ | 0.3 (0.2,  0.5) | 0.4 (0.2,  0.5) | 0.4 (0.2,  0.5) | 0.3 (0.2,  0.5) | 0.3 (0.2,  0.4) | 0.3 (0.2,  0.4) | _ | _ | _ | _ |
| GenR  (Netherlands) | 0.6 (0.4,  0.7) | _ | 0.5 (0.4,  0.7) | 0.5 (0.3,  0.6) | 0.4 (0.3,  0.6) | 0.4 (0.3,  0.5) | 0.4 (0.2,  0.5) | _ | _ | _ | _ |
| MoBa  (Norway) | 0.3 (0.2,  0.4) | _ | 0.4 (0.3,  0.4) | 0.4 (0.4,  0.5) | _ | _ | _ | _ | _ | _ | _ |
| The Raine study  (Australia) | _ | _ | 0 (‐0.3, 0.3) | 0 (‐0.2, 0.3) | 0.1 (‐0.1,  0.3) | 0.2 (0, 0.4) | 0.2 (0, 0.4) | 0.3 (0.1,  0.5) | _ | _ | _ |
| RHEA  (Greece) | _ | _ | _ | _ | 0 (‐0.5, 0.5) | 0.1 (‐0.4,  0.5) | 0.1 (‐0.3,  0.6) | 0.3 (‐0.2,  0.8) | _ | _ | _ |

Note: Model adjusted for maternal age birth. Values represent model‐predicted values. Predicted values shown from the youngest to oldest age of measurement.

Supplementary Figure 1: Slope index of inequality for internalising symptoms: examining linearity across levels of maternal education


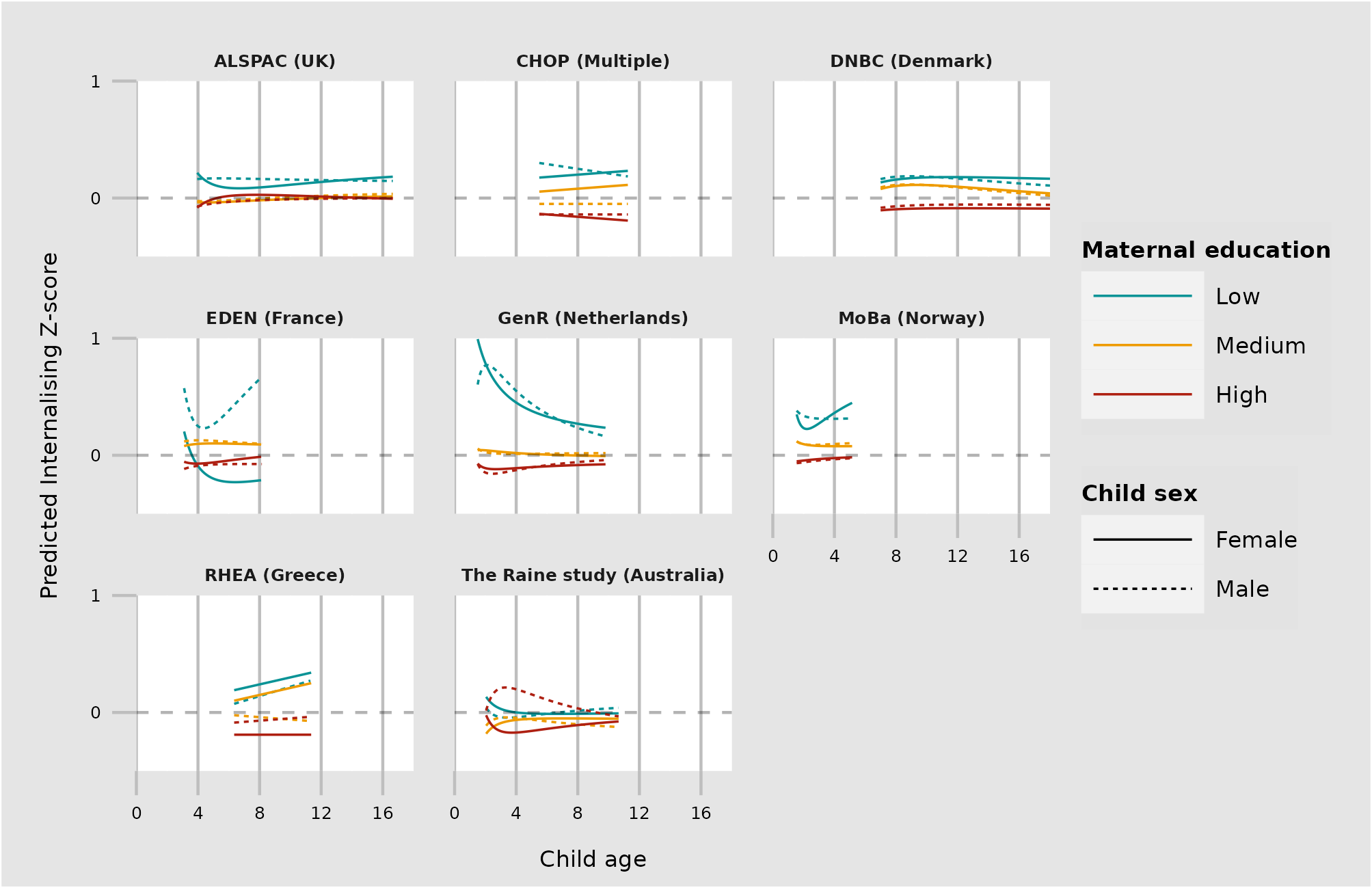


Note: Model adjusted for maternal age birth. Values represent model‐predicted values. Predicted values shown from the youngest to oldest age of measurement.

Supplementary Figure 2: Slope index of inequality for internalising symptoms: examining linearity across levels of maternal education


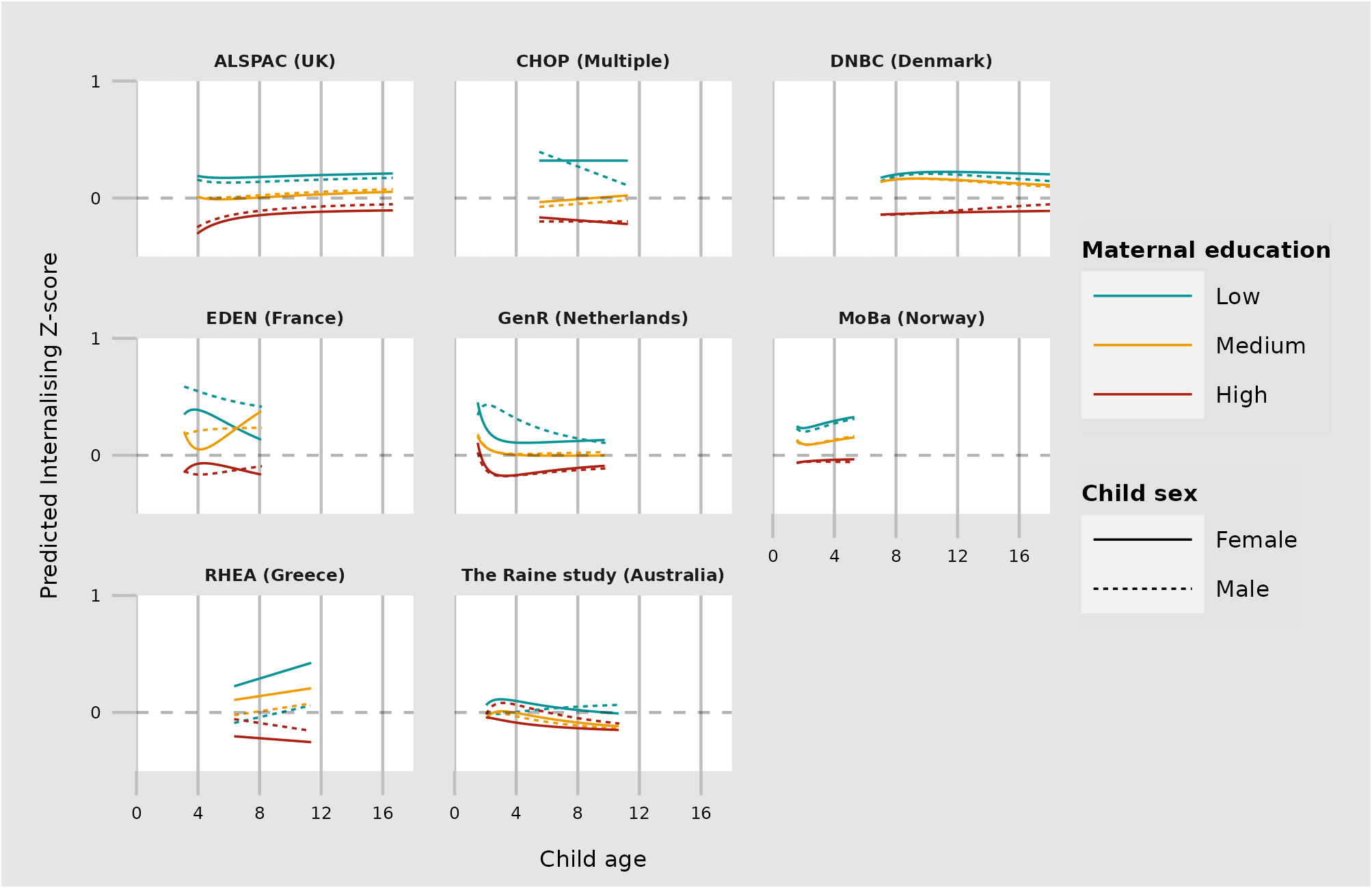


Note: Model adjusted for maternal age birth. Values represent model‐predicted values. Predicted values shown from the youngest to oldest age of measurement.

Supplementary Figure 3: Slope index of inequality for internalising symptoms based on household disposable income


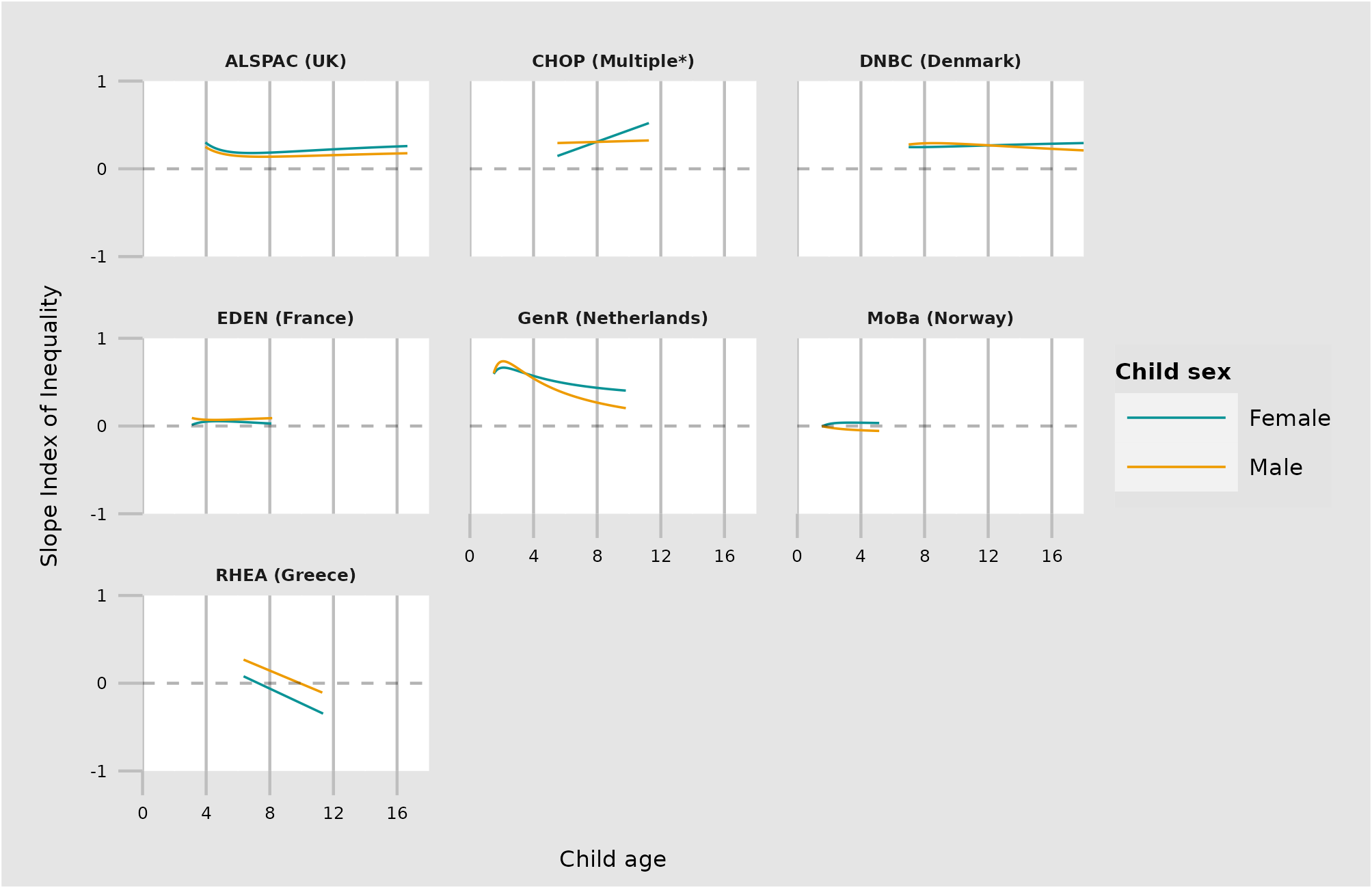


Note: Model adjusted for maternal age birth. Values represent model‐predicted values. Predicted values shown from the youngest to oldest age of measurement.

Supplementary Figure 4: Slope index of inequality for externalising symptoms based on household disposable income


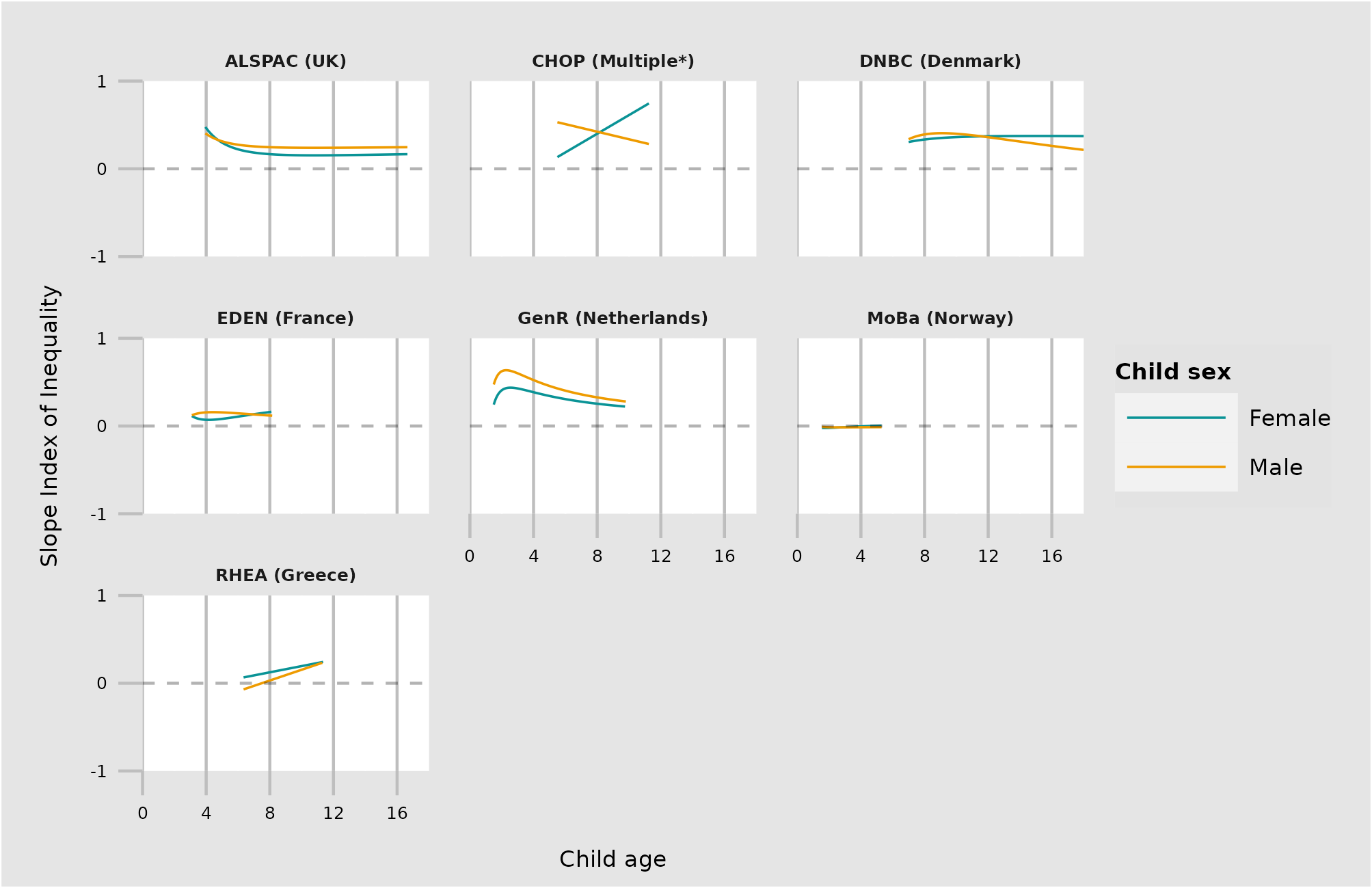


Note: Model adjusted for maternal age birth. Values represent model‐predicted values. Predicted values shown from the youngest to oldest age of measurement.

Supplementary Figure 5: Slope index of inequality for internalising symptoms comparing adjustment for ethnicity


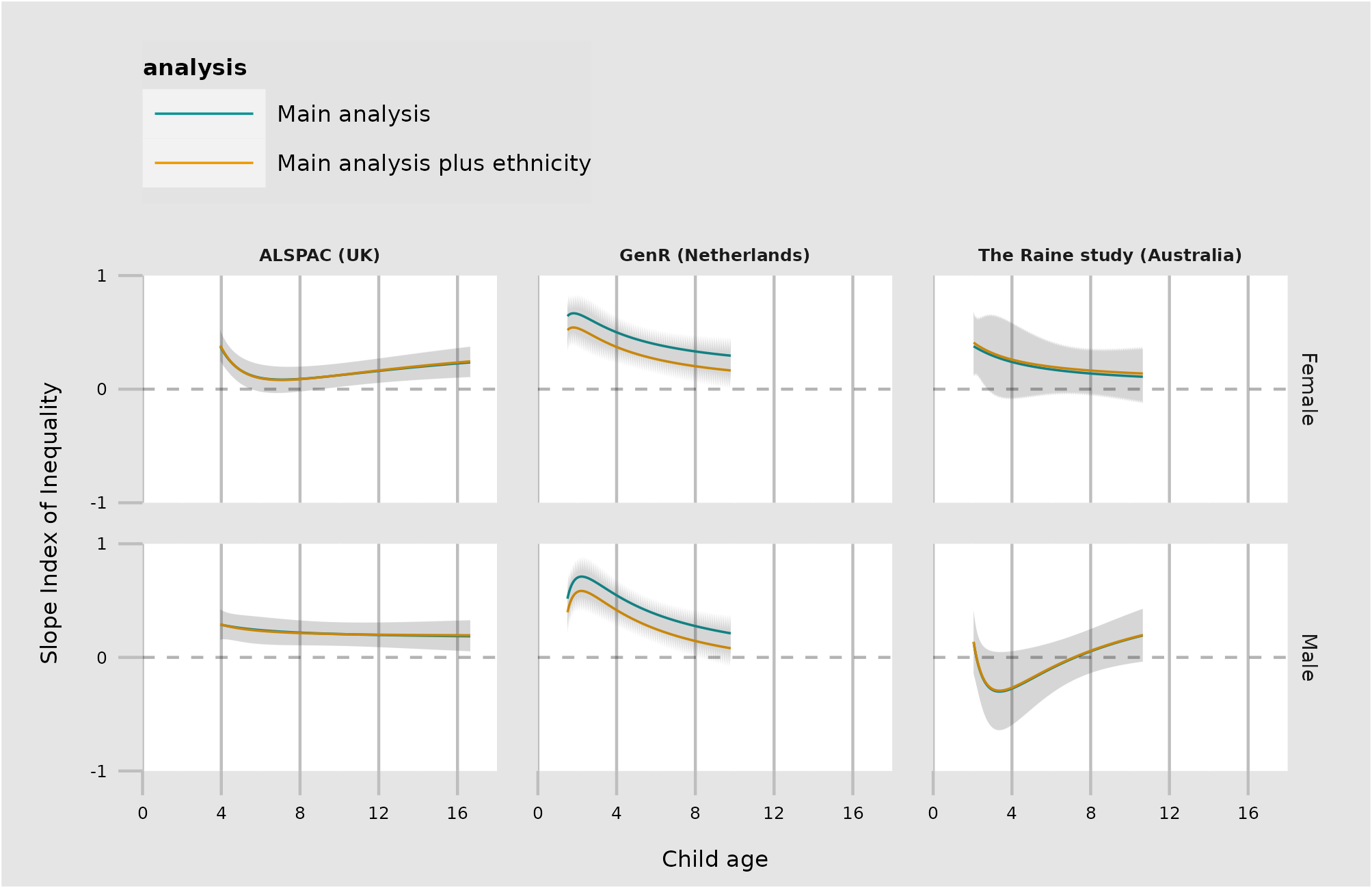


Note: Model adjusted for maternal age birth and maternal ethnicity. Values represent model‐predicted values. Predicted values shown from the youngest to oldest age of measurement.

Supplementary Figure 6: Slope index of inequality for externalising symptoms comparing adjustment for ethnicity


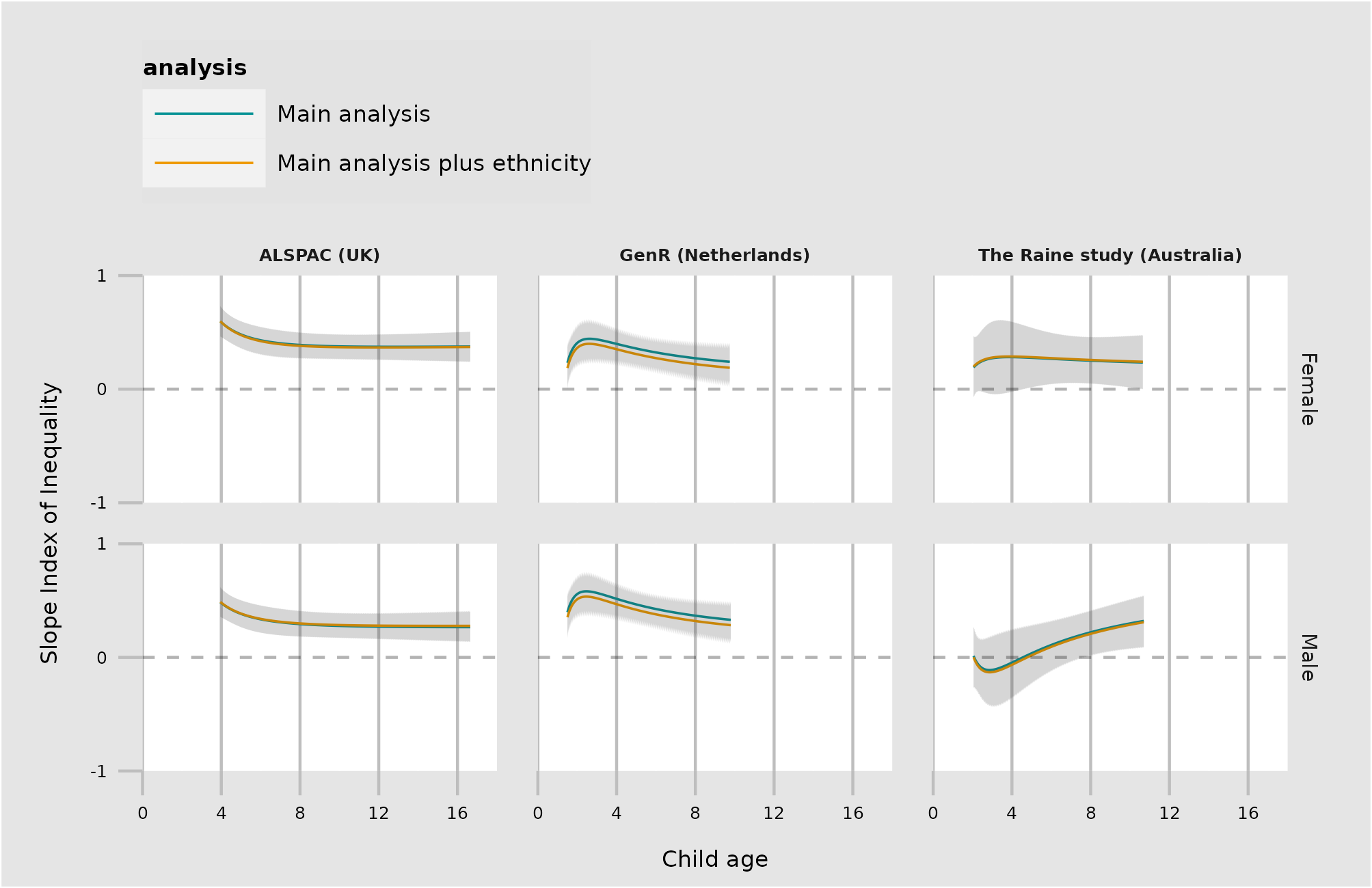


Note: Model adjusted for maternal age birth and maternal ethnicity. Values represent model‐predicted values. Predicted values shown from the youngest to oldest age of measurement.

Supplementary Figure 7: Slope index of inequality for internalising symptoms comparing adjustment for maternal mental health problems


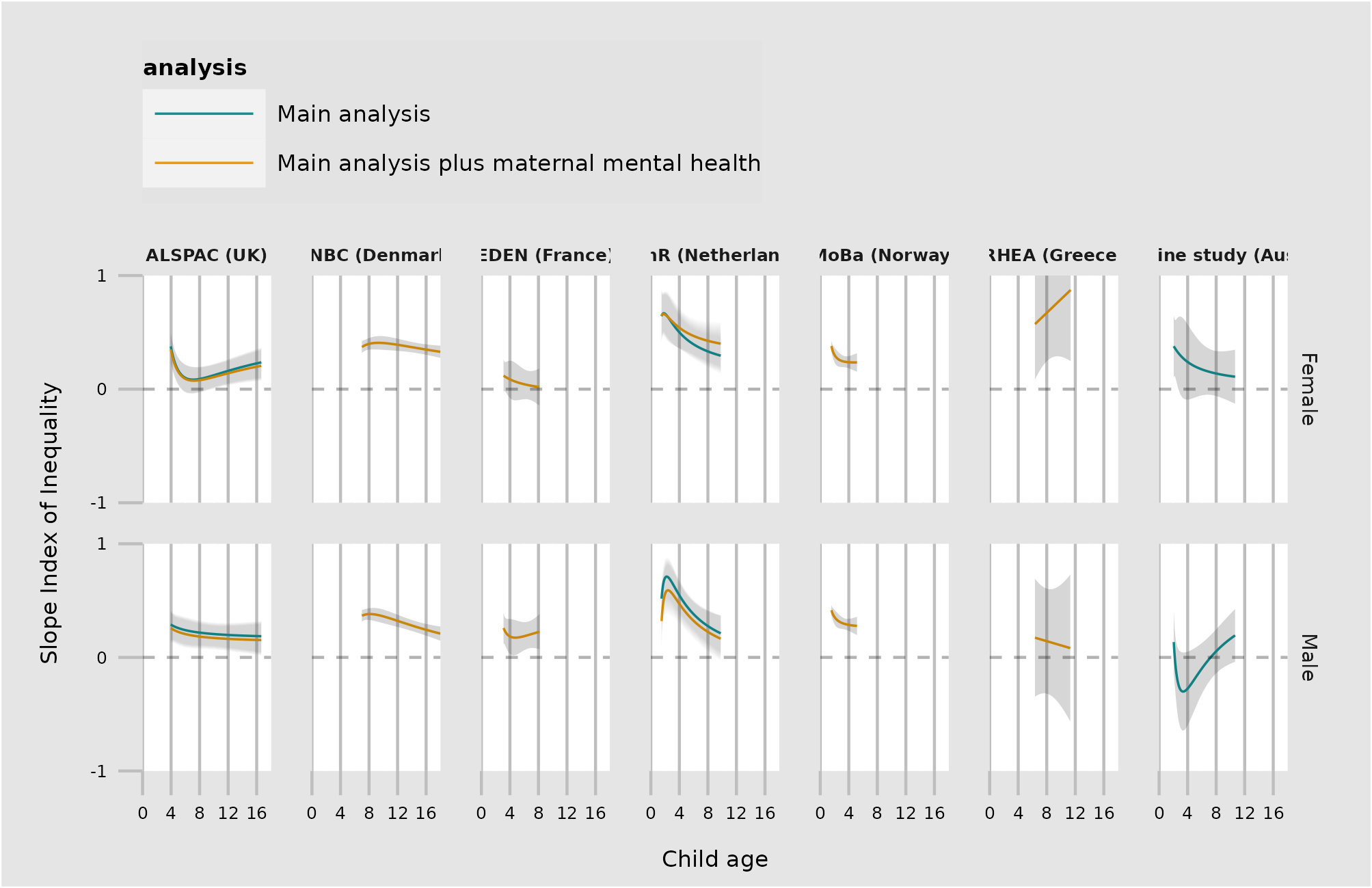


Note: Model adjusted for maternal age at birth and maternal pre‐pregnancy mental health problems. Values represent model‐predicted values. Predicted values shown from the youngest to oldest age of measurement.

Supplementary Figure 8: Slope index of inequality for externalising symptoms comparing adjustment for maternal mental health problems


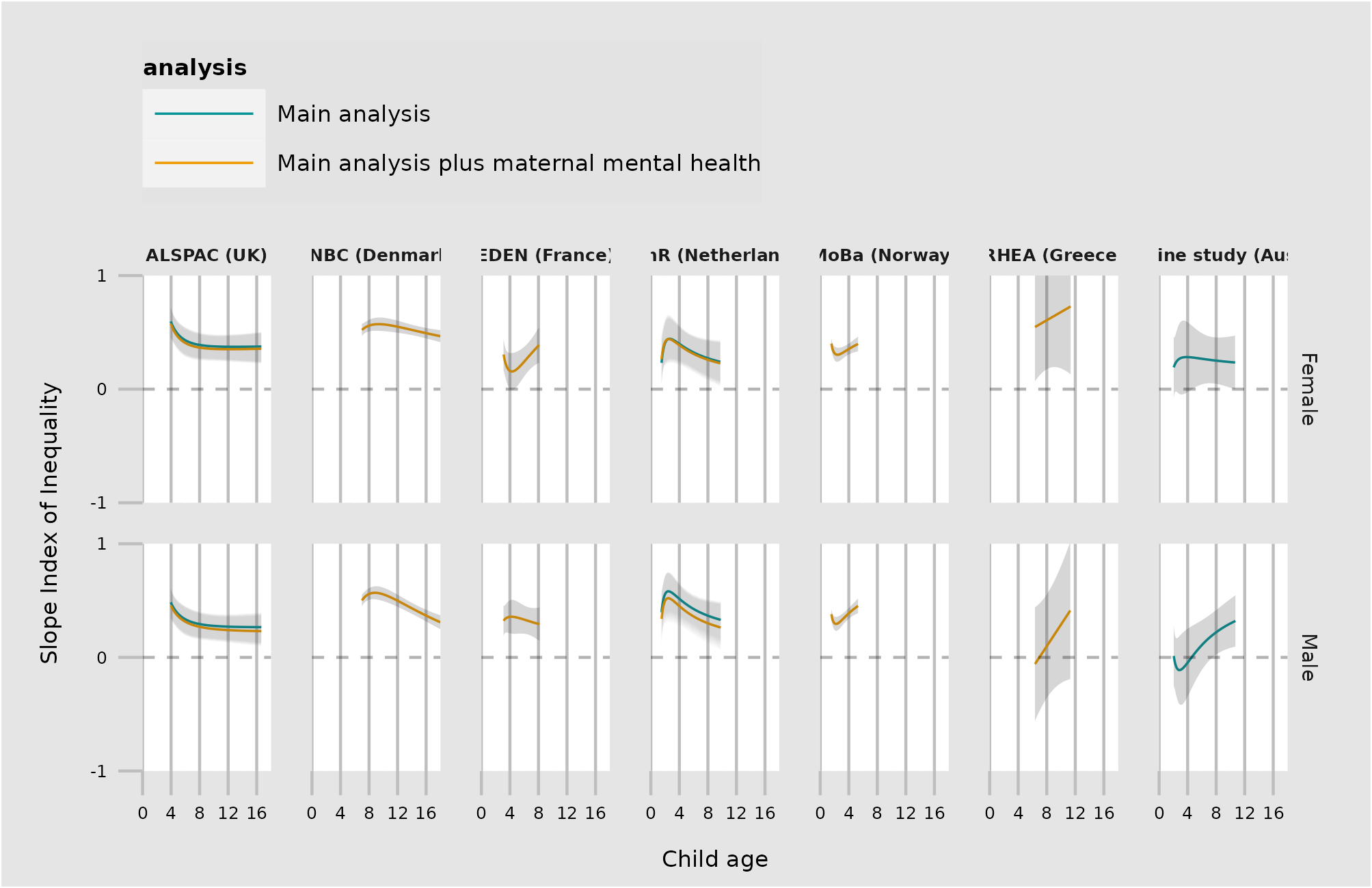


Note: Model adjusted for maternal age at birth and maternal pre‐pregnancy mental health problems. Values represent model‐predicted values. Predicted values shown from the youngest to oldest age of measurement.

# **References**

1. Andy Boyd et al. “Cohort profile: the ‘children of the 90s’—the index offspring of the Avon Longitudinal Study of Parents and Children”. In: *International journal of epidemiology*

42.1 (2013), pp. 111–127.

1. Abigail Fraser et al. “Cohort profile: the Avon Longitudinal Study of Parents and Children: ALSPAC mothers cohort”. In: *International journal of epidemiology* 42.1 (2013), pp. 97–110.
